# Interdigitated Columnar Representation of Personal Space and Visual Space in Human Parietal Cortex

**DOI:** 10.1523/JNEUROSCI.0516-22.2022

**Published:** 2022-11-30

**Authors:** Roger B.H. Tootell, Zahra Nasiriavanaki, Baktash Babadi, Douglas N. Greve, Shahin Nasr, Daphne J. Holt

**Affiliations:** ^1^Department of Psychiatry, Massachusetts General Hospital, Charlestown, Massachusetts 02129; ^2^Harvard Medical School, Boston, Massachusetts 02115; ^3^Athinoula A. Martinos Center for Biomedical Imaging, Massachusetts General Brigham Hospital, Boston, Massachusetts 02129; ^4^Department of Radiology, Massachusetts General Hospital, Charlestown, Massachusetts 02129

**Keywords:** 7T, cortical columns, high-resolution fMRI, personal space

## Abstract

Personal space (PS) is the space around the body that people prefer to maintain between themselves and unfamiliar others. Intrusion into personal space evokes discomfort and an urge to move away. Physiologic studies in nonhuman primates suggest that defensive responses to intruding stimuli involve the parietal cortex. We hypothesized that the spatial encoding of interpersonal distance is initially transformed from purely sensory to more egocentric mapping within human parietal cortex. This hypothesis was tested using 7 Tesla (7T) fMRI at high spatial resolution (1.1 mm isotropic), in seven subjects (four females, three males). In response to visual stimuli presented at a range of virtual distances, we found two categories of distance encoding in two corresponding radially-extending columns of activity within parietal cortex. One set of columns (P columns) responded selectively to moving and stationary face images presented at virtual distances that were nearer (but not farther) than each subject's behaviorally-defined personal space boundary. In most P columns, BOLD response amplitudes increased monotonically and nonlinearly with increasing virtual face proximity. In the remaining P columns, BOLD responses decreased with increasing proximity. A second set of parietal columns (D columns) responded selectively to disparity-based distance cues (near or far) in random dot stimuli, similar to disparity-selective columns described previously in occipital cortex. Critically, in parietal cortex, P columns were topographically interdigitated (nonoverlapping) with D columns. These results suggest that visual spatial information is transformed from visual to body-centered (or person-centered) dimensions in multiple local sites within human parietal cortex.

**SIGNIFICANCE STATEMENT** Recent COVID-related social distancing practices highlight the need to better understand brain mechanisms which regulate “personal space” (PS), which is defined by the closest interpersonal distance that is comfortable for an individual. Using high spatial resolution brain imaging, we tested whether a map of external space is transformed from purely visual (3D-based) information to a more egocentric map (related to personal space) in human parietal cortex. We confirmed this transformation and further showed that it was mediated by two mutually segregated sets of columns: one which encoded interpersonal distance and another that encoded visual distance. These results suggest that the cortical transformation of sensory-centered to person-centered encoding of space near the body involves short-range communication across interdigitated columns within parietal cortex.

## Introduction

The importance of personal space (PS) regulation has been highlighted recently by the widespread adoption of “social distancing” practices, designed to reduce COVID-19 transmission (Cartaud et al., 2020, Iachini et al., 2021; Welsch et al., 2020, 2021; Holt et al., 2022). However, well before the introduction of this deliberate social distancing, numerous behavioral studies have described a fundamental discomfort that people experience when an unfamiliar person becomes “too close,” intruding into their PS (Hayduk, 1978, 1983). This interpersonal distancing between conspecifics may be related to the survival-based distancing that occurs between prey and predator species (Hediger, 1955; Ardrey, 1966).

Most models of PS processing distinguish between an inner (personal) zone, and the distance beyond that. This inner zone extends from the body surface to the PS boundary, typically 60–100 cm away (Hayduk, 1983). Repeated measurements of the distance to the PS boundary are highly reliable within a given individual but vary substantially between individuals (Hayduk, 1983; Bar-Haim et al., 2002; Cléry and Hamed, 2018; Tootell et al., 2021; Welsch et al., 2021). Within this inner zone, discomfort increases gradually with closer interpersonal proximity (Hayduk, 1981a; Welsch et al., 2019; Tootell et al., 2021). Beyond the PS boundary, discomfort is typically not elicited by the presence of another person (but see Welsch et al., 2019). Similarly, skin conductance (a measure of sympathetic nervous system arousal) increases when an experimental confederate is positioned close to a subject within (but not beyond) the PS boundary (Candini et al., 2021; Tootell et al., 2021). The concept of PS (as defined by the discomfort and/or defensive response evoked by interpersonal intrusion) is closely related to the concepts of peri-personal space (PPS; Bogdanova et al., 2021), body ownership (BO), and body schema.

Relatively little is known about the brain mechanisms that underlie PS processing. Several human fMRI studies suggest a role for the parietal cortex in the processing of PS and/or BO (Lloyd et al., 2006; Schienle et al., 2015; Grivaz et al., 2017). One study (Holt et al., 2014) described higher fMRI activity in response to images of approaching (compared with withdrawing) faces, in two specific parietal regions. Because these responses were stronger to faces than objects, these results were interpreted as a selective response to a social stimulus, rather than to approaching stimuli per se.

Some involvement of parietal cortex in PS processing is not unexpected because: (1) PS intrusion is a specific type of threatening event, and (2) in monkeys, threat-defensive responses have been reported in parietal cortex (and in premotor areas F4/5, which are interconnected with parietal cortex; Luppino et al., 1999). For instance, defensive behavior was repeatedly elicited by electrical micro-stimulation in inferior parietal cortex (Cooke et al., 2003) and (at lower threshold) in areas F4/5 (Graziano et al., 2002).

Threats in the visuo-spatial environment might lead to transformations in distance encoding within the brain, to enhance the representation of the threatening stimuli. Thus, here we investigated whether an eye-centered spatial representation (from occipital cortex) is transformed into a body-centered (more egocentric, or PS-related) representation in inferior parietal cortex, as previously suggested (Hadjidimitrakis et al., 2014; Cléry et al., 2017).

Such a hypothetical transformation from eye-centered to body-centered spatial representations could be topographically organized in different ways, including: (1) a large-scale gradient distributed across the parietal surface, (2) in multiple smaller-scale local networks (e.g., cortical columns), or (3) randomly, without systematic topographic organization. Such alternative organizations imply correspondingly differing computational schemes. The current findings generally supported the second model. Our results demonstrated multiple, local transformations of spatial information in parietal cortex, mediated via two types of BOLD-defined radial groupings (“columns”). These two column types differed in the nature of their spatial encoding properties, from visual to more egocentric spatial scales. To our knowledge, no type of cortical column has been described previously in human parietal cortex.

## Materials and Methods

### Experimental design and statistical analysis

#### Participants

Seven human subjects (four females; age range: 22–29 years; mean: 24.6 years) participated in this study. This sample size was based on those of similar, prior studies of 7 Tesla (7T) fMRI data analyzed initially at the single subject level (Nasr et al., 2016; Tootell and Nasr, 2017; Nasr and Tootell, 2018a,b, 2020).

All subjects had normal or corrected-to-normal visual acuity (Snellen test) and radiologically normal brains, and were without any history of neuropsychiatric disorders. Written informed consent was obtained from all subjects before enrollment, in accordance with the Declaration of Helsinki. All experimental procedures were conducted according to Massachusetts General Hospital protocols, approved by the Massachusetts General Brigham Institutional Review Board. All data for this study were collected between February through October 2019, before the onset of the COVID pandemic in the greater Boston area.

#### Measurements of personal space

In each subject, the size of PS was measured in two ways. First, we used the standard stop distance procedure (SDP; Williams, 1971; Hayduk, 1978, 1983), in which each subject identified their preferred, comfortable distance from an “intruder” (an experimental confederate), in a neutral laboratory setting. During this procedure, subjects were asked to stand still, facing the intruder, who initially stood 3 m away from the subject. Subjects were instructed as follows: “Please stand still while my colleague walks slowly toward you. Say 'okay' when you start to feel slightly uncomfortable. When you say 'okay,' they will pause. Please make sure to maintain eye contact with my colleague throughout the procedure.” The distance between the subject and the intruder was measured after each trial, defined as the distance between the tips of each pair of shoes (the “stop distance”).

The second set of measurements was acquired while each subject lay prone in the MRI scanner, in the absence of scanning. Each subject performed a computerized version of the SDP, in which subjects were presented with face images which appeared to move toward them. A small central fixation target was superimposed on the bridge of the nose of each face image. The subjects were instructed to maintain fixation and to press a button on a button box when the face reached a virtual distance at which the subject began to feel uncomfortable.

The outside-the-scanner SDP values were generated as a standard for comparison to values in the literature, and to the within-scanner measurements. The within-scanner values were used to precalculate stimulus dimensions, and for the data analysis, and as a within-subject comparison to the outside-the-scanner values. In analyses of both types of PS measurement, the first trial was excluded as a “warm up” trial, as described previously (Hayduk, 1981b).

Our use of face images as stimuli here is consistent with previous studies that have also presented human-like stimuli, including mannequins, virtual reality-based avatars, and faces images (Bailenson et al., 2003; Mccall et al., 2009; Llobera et al., 2010; Rinck et al., 2010; Wieser et al., 2010; Iachini et al., 2014, 2016; Schienle et al., 2015; Ruggiero et al., 2016; Hecht et al., 2019; Welsch et al., 2019; Tootell et al., 2021), which can elicit PS-related responses analogous to those evoked by real human subjects (Tootell et al., 2021).

#### Visual stimuli

**Figure 1.**
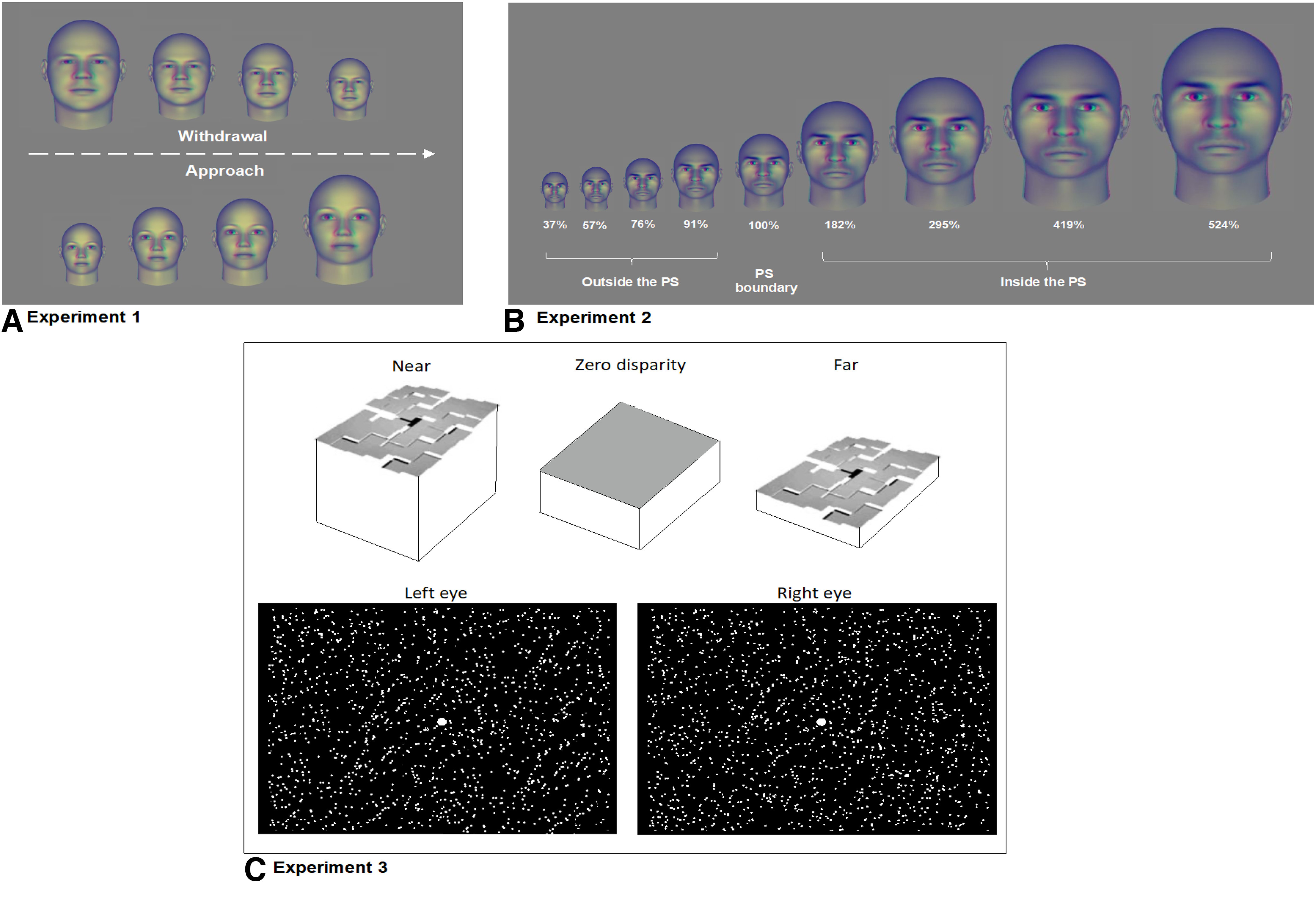
Schematic of the experimental paradigms used in the three experiments. ***A***, Experiment 1. Subjects were shown continuously approaching faces (in one 15-second block) or continuously withdrawing faces (in other 15 sec blocks), presented in pseudorandom order. Variations in virtual stimulus distance were calculated as co-variations in the size and disparity cues (disparity cues not shown here), calculated to match those in real life. Thus, the approaching and withdrawing stimuli were identical to each other, other than the motion direction. BOLD responses were corrected for the known hemodynamic delay, then subtracted from each other in subsequent analyses to identify the cortical sites in which stimuli were significantly biased toward the approaching or withdrawing stimuli. ***B***, Experiment 2. Subjects were shown stationary 3D faces at a range of virtual distances, using an event-related design (average stimulus duration = 3 seconds and ITI = 3 to 18 seconds) for each face distance. The virtual face distances ranged from 37% to 524% of each subject's individual personal space size, based on prior behavioral measurements. Presentation order was pseudorandomized. ***C***, Experiment 3. Subjects were presented with random dot stimuli (RDS), to test for (and localize) disparity selective columns. The top row shows the shape of the 3D stimuli evoked during successful stereoscopic fusion of the RDS stimuli. The surface topography of the cuboids is illustrated as in Tsao et al., 2003. The shapes evoked selective activation by 24-second blocks of dynamic disparity-based 'near' or 'far' distance, or (as a control condition) zero disparity in the center of gaze (i.e., a fronto-parallel plane). An example of the monocular stimuli (which were binocularly fused during the experiment) are shown. Binocular fusion was achieved using color anaglyphs. The central white dot is a fixation target.

Stimuli were presented via an LCD projector (1024 × 768-pixel resolution, 60-Hz refresh rate) onto a rear-projection screen, viewed through a mirror mounted on the receive coil array. MATLAB 2018b (MathWorks) and the Psychophysics Toolbox were used for stimulus presentation (Brainard, 1997; Pelli, 1997). Details of the experiments are described below, and schematized in Figure 1.

#### Experiment 1: approaching versus withdrawing 3D face stimuli

A series of sixteen face images were presented, in four different conditions: Female Approach, Female Withdrawal, Male Approach, and Male Withdrawal. These face stimuli had emotionally neutral (no valence) expressions. All faces were generated using a commercially available program (FaceGen), based on a customizable 3D face mesh. Multiple facial features, including skin pigmentation and face morphology, were customizable on the mesh surfaces, and varied across the set of faces. The face stimuli included 6 Caucasian, 2 Asian, 4 African-American, and 4 Multi-Racial -appearing face images. Examples of these face images are shown in Figure 1*A*,*B*. Each approaching or withdrawing face series was presented throughout a single block (15 s in duration), in a pseudorandom order, using eighteen blocks/run, and eight runs/session. To define baseline BOLD levels, two 15-s displays of uniform gray (including a small central fixation target) were also presented, one at the beginning and one at the end of each run.

The experimental manipulation of virtual distance was based on differences in face size and binocular disparity, presented within a wide field of view, to match the corresponding visual changes that would occur during the perception of a real person, either approaching or withdrawing, across a range of 39–300 cm from the viewer, at a constant walking speed (1.12 m/s). Consistent with published values, we assumed an average real-world face width of 16 cm. Within the scanner, these synthetic 3D faces were viewed at a real distance of 114 cm from the screen (i.e., the sum of distances from eye to mirror, plus from the mirror to the stimulus screen), thus 32 cm on the stimulus screen. The mean (real and virtual) PS size (i.e., 100% of the average virtual PS size) were both ∼60 cm from the viewer, thus ∼33.6 cm on the stimulus screen. (Because the size of PS varied between individuals, these values are only illustrative.)

The stimuli were similar to those used previously (Holt et al., 2014), except that 3D cues were added to the face images, based on real-world differences in binocular disparity at different viewing distances. Subjects viewed all disparity-varying stimuli using red-cyan anaglyph glasses [Kodak Wratten filters no. 25 (red) and no. 44A (cyan)] that were mounted on the top of the receive head coil, inside the scanner. Otherwise, virtual face distance was made as realistic as possible. For instance, in real life, binocularly viewed variations in face distance include small 3D face rotations, in opposed directions, in each of the component monocular views. Such realistic image changes were incorporated in the 3D face images used here. We also assumed an interpupillary distance of 6 cm. Thus, when viewed at 57 cm, an interpupillary disparity of 6 degrees in the 3D mesh would be matched by a rotation of the face mesh by three degrees in opposite rotation directions, for each eye. At different viewing distances, these values differed accordingly.

Subjects performed a dummy attention task during presentation of the face stimuli, in which a colored dot was presented repeatedly within each block at a random location on the computer screen (three presentations/block, each with a duration of 400 ms, timed unpredictably relative to the main stimuli). Subjects were instructed to maintain fixation on a small target located at the center of the images (specifically, at the junction between the bottom of the forehead and the upper terminus of the nose), and to press a button (located on the button box inside the scanner) whenever they saw the dot presented on the screen. The instructions were repeated before each run.

#### Experiment 2: stationary 3D face stimuli

In experiment 2, subjects viewed stationary face images, using a subset of the images that were presented during experiment 1. Here, each stationary face was presented at different virtual distances, in a pseudorandom order, in an event-related design. Each face was presented for 3 s followed by an intertrial interval (a spatially uniform gray screen) varying between 3 and 12 s. Virtual face distances were individually precalculated for each subject, based on each subject's PS boundary as measured previously within the scanner. Each face was presented at nine systematically varied virtual distances: four distances inside the subject's PS boundary, four distances outside the subject's PS boundary, and one distance corresponding to the subject's PS boundary (i.e., at 37%, 57%, 76%, 91%, 100%, 182%, 295%, 419%, and 524% of each subject's PS boundary).

#### Experiment 3: binocular disparity (stereoscopic) stimuli

In this experiment, disparity-varying stimuli were presented in sparse (5%) random dot stereograms based on small red or green squares (each 0.09° × 0.09°) presented against a black background, which extended 20° × 20° in the visual field. Two random dot stimuli (each either red or green) were overlaid on the display screen, and binocularly fused during BOLD acquisitions. In the main condition, stimuli formed a stereoscopic percept of a regular array of cuboids that varied sinusoidally in-depth, i.e., within 0.2–0.4° of horizontal binocular disparity, stereoscopically either “near” or “far,” relative to the fixation plane, with independent phase, similar to stimuli described earlier (Tsao et al., 2003; Nasr et al., 2016; see Fig. 1*C*). In the control condition, the fused percept spanned a fronto-parallel plane intersecting the fixation target (i.e., zero depth at the point of ocular convergence). Each block-designed experimental run included eight stimulus blocks (24 s/block), beginning and ending with an additional 12 s of uniform gray (“blank”). When viewed monocularly, or when the binocular images were identical, these control stimuli instead appeared as an array of dots, moving coherently and slowly from left to right (and vice versa) in the zero disparity plane (uncorrected for variation in the arc tangent).

To measure and control the level of attention during the 3D disparity scans, subjects were required to report changes in the shape (identifying the longer axis) of a small (average length/width = 0.1–0.2°) rectangular fixation target, by pressing a button located on the button box inside the scanner.

### Imaging

All MRI experiments were conducted in a 7T Siemens whole-body scanner equipped with SC72 body gradients (maximum gradient strength, 70 mT/m; maximum slew rate = 200 T/m/s) using a 32-channel helmet receive coil array and a birdcage volume transmit coil. The anatomic data collected from each subject was based on a high-resolution multi-echo T1-weighted magnetization-prepared gradient-echo image [MPRAGE, 0.80-mm isotropic voxels, with a repetition time (TR) of 2530 ms, an inversion time of 1100 ms, an echo time (TE) of 1.76 ms, a flip angle (FA) of 7° and a field of view (FOV) of 240 mm]. The MR sequence used in this study did not include any parallel or partial Fourier imaging techniques. Echo spacing was 0.79 ms (no free echo spacing). Functional images were acquired using a single-shot gradient-echo EPI with 1.1 mm (isotropic) voxel size, TR = 3000 ms; TE = 26 ms; flip angle = 90°; FOV = 192 mm, number of slices = 51; EPI factor = 174. Each run included 90 TRs in experiments 1 and 2, and 80 TRs in experiment 3. In all scans, the coverage included the dorsal and posterior occipital cortex, and dorsal parietal cortex [see region of interest (ROI)]. In each subject, the number of functional volumes acquired was 1440 for experiment 1, 720 for experiment 2, and 1280 for experiment 3.

### General imaging data analysis

All structural and functional MRI data analyses were performed using the FreeSurfer Functional Analysis Stream (FSFAST) v.6 (https://surfer.nmr.mgh.harvard.edu/fswiki/FsFast). In four main steps, this included: (1) bias field correction, (2) cortical reconstruction, (3) preprocessing of the functional data, and (4) first level and group statistical analyses (Dale and Sereno, 1993; Dale et al., 1999; Fischl and Dale, 2000; Fischl et al., 2001, 2002, 2004a, b). Details of these steps are as follows.

#### Bias field correction

To correct the anatomic scan for signal intensity variation across the brain volume caused by inhomogeneity of the B1 field at 7T, a bias field correction was performed using SPM 8. A Bayesian model estimated a smooth function that was multiplied with the image using prior knowledge about the field distributions likely to be encountered (Ashburner and Friston, 2005). The model assumed that the field inhomogeneity (a type of “noise”) is multiplicative and because of variations of the tissue properties in each voxel, rather than because of noise from the scanner. The full-width-half-maximum (FWHM) for the Gaussian smoothness used a bias of 18 mm, with a very light (0.0001) bias regularization (Zaretskaya et al., 2018).

#### Cortical reconstruction

The automated reconstruction of the structural data (FreeSurfer; https://surfer.nmr.mgh.harvard.edu) included the following steps: image realignment and motion correction, skull stripping (removing the nonbrain tissue), gray-white matter segmentation, reconstruction of cortical surface models, and labeling of the regions on the cortical surfaces based on probabilistic information, derived from a manually labeled training set.

#### fMRI preprocessing

BOLD data were registered to the same-subject anatomic data using boundary-based registration (Greve and Fischl, 2009) and corrected for head motion, then sampled onto the cortical surface, where all further analysis was performed. Data were not spatially smoothed (i.e., 0 mm FWHM). The hemodynamic response amplitudes for each condition were estimated using a general linear model in FSFAST. Regressors were constructed by convolving the stimulus box car with the SPM canonical hemodynamic response function. Head motion parameters were treated as nuisance regressors; polynomial regressors were included to account for low-frequency drift. Voxel-wise statistical tests were conducted by computing functional contrasts.

During this analytical step, we measured and corrected for head motion and EPI distortion using two programs in FreeSurfer. First, the tkregister2 program allowed us to visually check for possible deviations in the otherwise-automated registration of functional to anatomic alignment. In very few cases, we found minor deviations in the automated registration, which were corrected manually.

Second, we used “tkregister-sess” to check the registration cost values. These cost values increased between 0 and 1. For this study, values below 0.7 were considered acceptable (https://www.freesurfer.net/pub/dist/freesurfer/tutorial_versions/freesurfer/fsfast/bin/tkregister-sess). In our experimental total of 264 runs (eight runs/session, and 33 sessions), the registration cost values across all/each of these runs were significantly lower than 0.7 (mean cost value = 0.25, SD = 0.13).

#### Quality control

All brain images were visually inspected for brain coverage, registration quality and anatomic defects. The FSL motion outlier algorithm was used to eliminate time points showing excessive head motion across volumes (Power et al., 2012), by using a compound matrix of outlier timepoints as a regressor in the general linear model (GLM). Among the several metrics available for defining outlier time points (Power et al., 2012; Bastiani et al., 2019), we used the DVARS metric (D referring to temporal derivative of time courses, VARS referring to RMS variance over voxels; Smyser et al., 2010), which reflects the rate of change of the BOLD signal across the entire brain in comparison to the previous time point (Power et al., 2012). The threshold used to define an outlier was set to the default value suggested by Power et al. (2012; 75th percentile + 1.5 times the interquartile range of the DVARS metric). Any time point with a value larger than this threshold was eliminated from further analysis, as an outlier. Very few timepoints (0.08% of the total) were rejected based on this criterion.

In addition to time point motion correction, the six rigid-body motion parameters within each volume were used as regressors, to account for absolute motion. Based on this threshold criterion, 9.9% of the timepoints were excluded, across all the runs (*n* = 264) from all three experiments.

In the responses to the dummy attention task, outliers were defined as a score (in any of the experiments) of less than two standard deviations from the mean. No data met this criterion (experiment 1: M = 92.15%, SD = 3.3; experiment 3: M = 93.48%, SD = 7.09).

#### Region of interest (ROI)

In prior group-averaged, conventional-resolution fMRI studies, preferential activity to both approaching/withdrawing (Holt et al., 2014) and disparity-selective (Tsao et al., 2003) stimuli were found within the dorsal medial and lateral banks of the intraparietal sulcus (IPS). Accordingly, the ROI used here was defined based on anatomic (sulcal/gyral) features bounding this region, specifically including the dorsal half of the dorsal parietal-occipital sulcus (dPOS) on the medial bank, continuing to the lateral bank of the IPS, then crossing perpendicularly to the small sulci on the lateral bank of the IPS, to intersect the starting point near dPOS. In the single-subject data, this ROI was defined on the cortical surface of each subject. In the group averaged data, this ROI was defined based on the averaged gyri and sulci in the group-averaged cortical surface (Fischl et al., 1999). This ROI includes areas POIPS, VIPS, hLIP, and VIPS as previously described (Orban et al., 2006), and perhaps areas SPL 7A and 7P (likelihood 45% and 32%, respectively) of Julich and colleagues (Amunts et al., 2020).

### Specific analysis of 7T data

#### Consistency across sessions

To quantify the level of consistency between approach-biased and withdrawal-biased activity across different scan sessions, we measured the fMRI signal change evoked by the functional contrasts of interest, at a threshold of *p* < 0.05 (Face Approach > Face Withdrawal and Face Withdrawal > Face Approach), across the first two scan sessions of each experiment, for each vertex within the ROI, and in the average of the two scan sessions. The level of overlap in the resultant maps was used to quantify the consistency across sessions, when aligned (100% overlap = perfect consistency), compared with when systematically nonaligned. These control measurements of nonaligned maps were calculated by pseudo-randomizing the alignment of maps from session 1 relative to session 2.

#### Consistency across depth

To assess the radial (“columnar”) consistency in BOLD-activated sites, we subdivided and compared fMRI activity centered at multiple cortical depths, as described previously (Polimeni et al., 2010). For each subject, one surface was generated at the interface between the gray matter and the white matter (depth = 1.0), and a second surface was generated at the superficial boundary between the gray matter and the pia (depth = 0.0), based on each subject's high-resolution structural scans using FreeSurfer (Dale et al., 1999; Fischl et al., 2002). In addition to these two bounding surfaces, intermediate “mid-gray” surfaces were also generated, e.g., at 50% of the depth of the local gray matter (depth = 0.5, including cortical layer 4), and at 80% of the depth of the local gray matter (depth = 0.8, including layers 5 and 6; Dale et al., 1999).

#### Activity maps from experiment 1 (approach/withdrawal)

To create group-averaged maps, cortical surfaces from all hemispheres and subjects (14 hemispheres) were registered to an average reference surface for each hemisphere, and functional data were registered onto the average anatomic data, corrected for head motion and normalized without spatial smoothing (FWHM = 0; Fischl et al., 1999). Statistical analyses were performed by fitting a univariate general linear model (GLM) with a one-sample group mean design (OSGM), using a weighted least square to the fMRI data. The significance maps were created based on the predefined conditions, at a threshold of *p* < 0.01.

#### Columnar organization

To examine the BOLD maps from a viewpoint perpendicular to the cortical topography, the BOLD signal change evoked by the functional contrast of interest (face approach vs face withdrawal) was sampled at multiple equally-spaced points across the cortical depth. That activity was then illustrated along a line drawn along each subject's cortical surface map, which crossed patches of high and low activity.

Next, a more comprehensive analysis was conducted to test for a columnar organization across all subjects, within all parietal ROIs, by calculating the correlation between the selective activity in vertices distributed along each of the three cortical axes in the flattened cortical maps: X and Y within the surface-parallel cortical map, and Z along the perpendicular (radial) axis. As a control, we also measured BOLD activity at two different cortical depths: superficial (depth = 0.1) and deep (depth = 0.9). Given the cortical thickness within the ROI (mean = 2.37 mm), corresponding points on these two surface maps were centered 1.9 mm apart in depth, on average. For each of the three axes, correlation values were then calculated independently for each subject, and compared using paired *t* tests following transformation of the Pearson *r* values to z values using Fisher's transformation formula.

#### BOLD responses from experiment 2 (stationary faces)

For each subject, the average BOLD responses within the approach-selective and withdrawal-selective columns were calculated for each virtual stimulus distance. These distances were calculated separately for each subject, as percentages of the same subject's mean PS boundary, measured in the scanner.

#### Overlap measurements of the approach/withdrawal versus disparity-based activity maps

In several analyses, we measured the extent of overlap in evoked activity in different functional contrasts. For instance, we measured the overlap in the maps from each of four functional contrasts (approach-biased, withdrawal-biased, disparity near-biased and disparity far-biased), independently in the approach/withdrawal and disparity-selective columns, across a wide range of thresholds [log (p) 1.3 −10]. The overlap was calculated as a percentage of the surface area, in two ways: (1) when the maps were correctly aligned, and (2) (as a control condition) when one of the maps was randomly misaligned relative to the other (i.e., spatially shuffled) 1000 times.

Additional details of these experiments are equivalent to those published previously (Nasr et al., 2016; Tootell and Nasr, 2017, 2021; Nasr and Tootell, 2018a,b, 2020).

### Data/codes/software

Behavioral and brain data (available on request).

MATLAB (RRID: SCR_001622; https://www.mathworks.com).

FreeSurfer (RRID:SCR_001847; https://surfer.nmr.mgh.harvard.edu/fswiki/FsFast).

SPM (RRID:SCR_007037; https://www.fil.ion.ucl.ac.uk/spm/).

SPSS (RRID:SCR_007037; https://www.fil.ion.ucl.ac.uk/spm/).

Psychophysics Toolbox (RRID:SCR_002881; http://psychtoolbox.org/docs/Psychtoolbox).

## Results

### Behavioral measurements of personal space size

**Figure 2.**
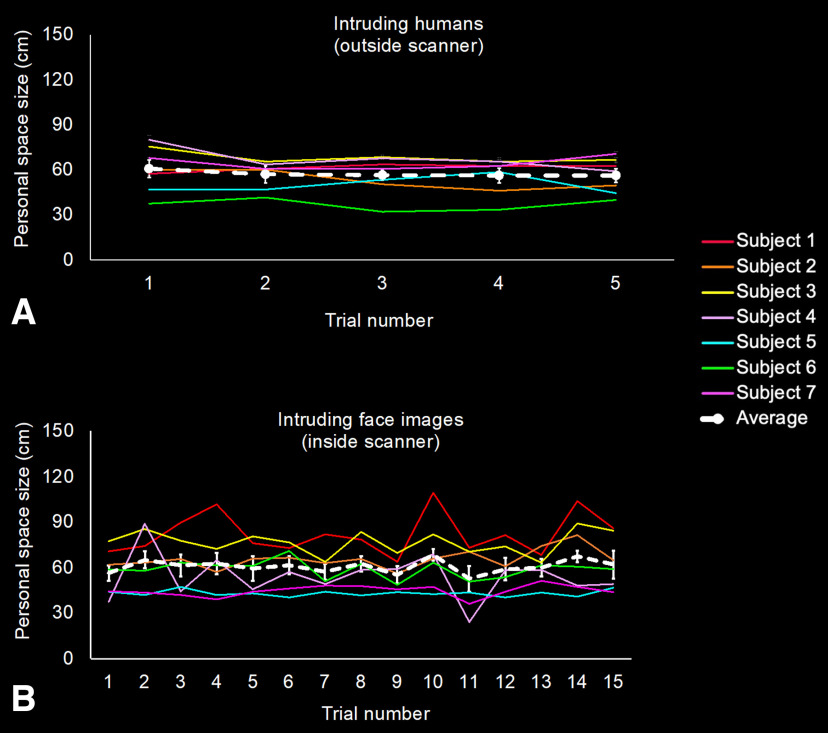
Personal space measurements differed between subjects but remained consistent across repeated measurements within each subject. ***A***, Measurements acquired outside the scanner, using the standard passive stop distance procedure, in response to real human confederates (“intruders”). ***B***, Analogous measurements to intruding face images, acquired while subjects were in the 7T scanner, in the absence of MR imaging. After a practice trial, personal space was measured in 5 (***A***) or 15 (***B***) trials, in each subject (*n* = 7) who was subsequently scanned in the main experiments. In both panels, data from the same subject are shown in a different, arbitrarily assigned color. The means are superimposed as a dashed white line. Error bars represent 1 SEM.

Figure 2 shows the PS size measurements for each subject using: (1) the standard stop distance procedure (SDP; Williams, 1971; Hayduk, 1981a, 1983), in response to an approaching human confederate outside of the scanner, and (2) in a within-scanner SDP, in response to the virtually-approaching 3D face images (see Materials and Methods). A random effect repeated measures ANOVA showed no significant differences among the SDP measurements collected for each subject (Greenhouse–Geisser *F*_(2.5,16.1)_ = 0.95, *p* = 0.45; mean = 57 cm, SD = 11.3; Fig. 2*A*). Similarly, in the within-scanner PS data, a repeated measures ANOVA showed statistically equivalent mean values over repeated measurements (Fig. 2*B*), i.e., without evidence for habituation across trials (Greenhouse–Geisser *F*_(2.9,17.8)_ = 1.85, *p* = 0.17; mean = 60.72 cm, SD = 15.01).

These behavioral results are consistent with results from prior studies which reported high within-subject reliability over time in the standard SDP (Hayduk, 1981a, 1983; Wormith, 1984; Tootell et al., 2021; Welsch et al., 2021). This demonstration of high reliability in the PS measurements was an important prerequisite for the subsequent analyses, insofar as our statistical sensitivity relied on extensive signal averaging over repeated measurements.

### Within-scanner task response accuracy

Mean accuracy during the dummy attention task (all subjects, all sessions) was 92.2% in response to the face stimuli, and 93.5% in response to the random dot stimuli.

### Experiment 1: approach-biased activity

In a prior study based on group-averaged (*n* = 22) conventional fMRI results (3T, 3 mm isotropic), presentation of approaching versus withdrawing face images consistently produced two patches of approach-biased activity in dorsal parietal cortex, one located posterior to the other (Holt et al., 2014). Using similar (but 3D) face stimuli here (see Materials and Methods), we tested for comparable maps of activity using a 7T scanner, in both group-averaged (*n* = 7) and in single subject analyses, at high (1.1 mm isotropic) spatial resolution.

**Figure 3.**
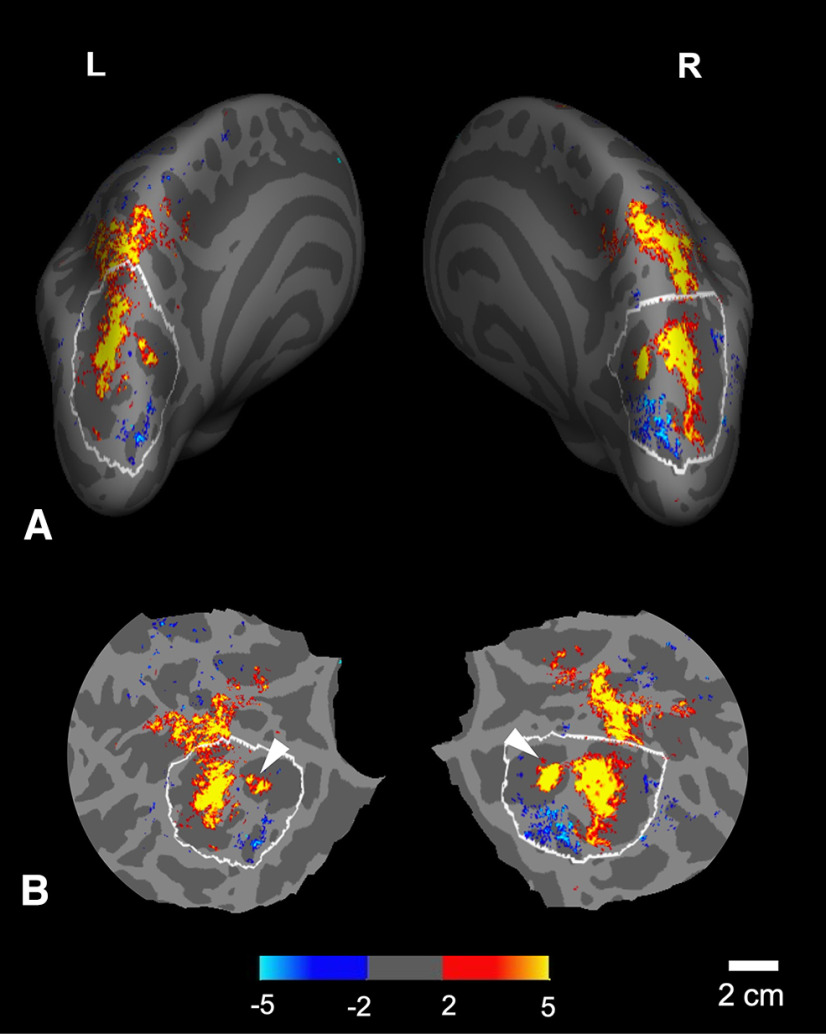
Group-averaged (*n* = 7) cortical activity in response to virtually approaching (compared with withdrawing) face images, scanned at 7T, 1.1 mm isotropic. Data are shown from left and right hemispheres (L and R, top) in the corresponding cortical surfaces, viewed from a posterior-dorsal viewpoint in inflated cortical format (***A***), and in a cortically flattened view (***B***). The significance scale on the bottom indicates -log10(p), where p is chance probability (yellow/red = approach > withdrawal; cyan/blue = withdrawal > approach). The ROI here (see white outlines, in posterior parietal cortex) was based on the averaged gyral/sulcal anatomy in the group-averaged cortical surface. In ***B***, the white arrows indicate clusters of activity within the dorsal parietal-occipital sulcus (dPOS). The spatial scale bar (bottom right) applies to the flattened view, subject to the expected distortion (±15%) due to cortical flattening.

Consistent with the earlier 3T results, the activity evoked here included two regions of approach-dominated activity, in anterior and posterior sites in the parietal cortex (Fig. 3). In the subsequent analyses, we focused on the posterior site, which was located in the posterior parietal cortex immediately anterior to (and known to share connections with) dorsal occipital (“visual”) cortex. Based on its proximity to visual cortex, we hypothesized that this posterior parietal ROI would be likely to show evidence for a transformation from eye-based spatial encoding (from visual cortex) to body-based spatial encoding (in parietal cortex).

A prior 3T study (Quinlan and Culham, 2007) found that a discrete site within the dorsal parietal-occipital sulcus (dPOS) responded selectively to “near” (compared with “far”) visual objects. Here, the corresponding parietal cortex site (Fig. 3*B*) also showed a strong bias for approaching (compared with withdrawing) stimuli, evident in the group-averaged activity, and in 11 (of 14) hemispheres tested.

### Single subject maps

**Figure 4.**
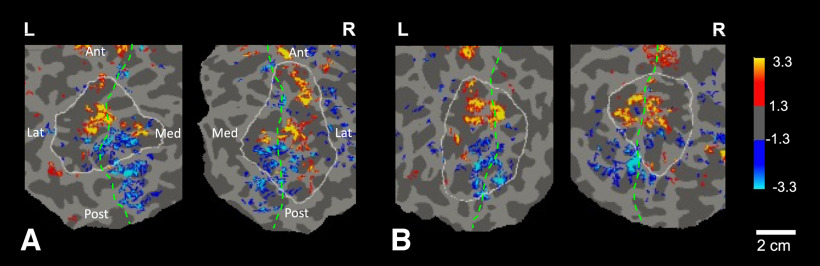
High-resolution approach vs. withdrawal activity maps from single subjects. Panels show flattened maps of cortical activity, in both hemispheres, in each of two individual subjects (1 and 3) in ***A*** and ***B***, respectively. The maps display approach-biased versus withdrawal-biased activity, in red-yellow and blue-cyan, respectively (scale bar on the right). The medial-lateral border (where the cortex folds) is shown as a dashed green line, and the parietal ROI is indicated by a white outline. Ant, anterior; Post, posterior; Med, medial; Lat, lateral; L, left hemisphere; R, right hemisphere. The significance scale indicates -log10(p).

**Figure 5.**
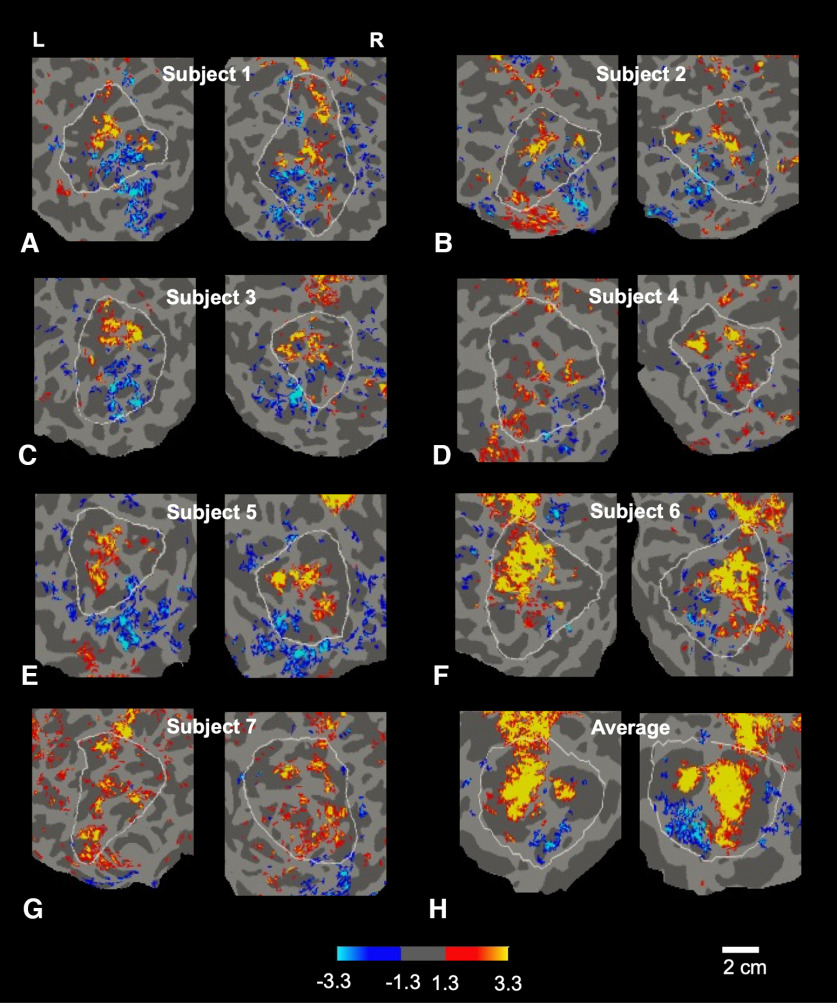
Response to approaching versus withdrawing face stimuli in each subject. Approach-biased versus withdrawal-biased activity is displayed in red-yellow and blue-cyan, respectively. The significance scale indicates -log10(p). All maps were averaged across both scan sessions, in each individual subject, in both right and left hemispheres (***A–G***), in the parietal cortex ROI (indicated by white outlines). ***H***, Average across all subjects.

**Figure 6.**
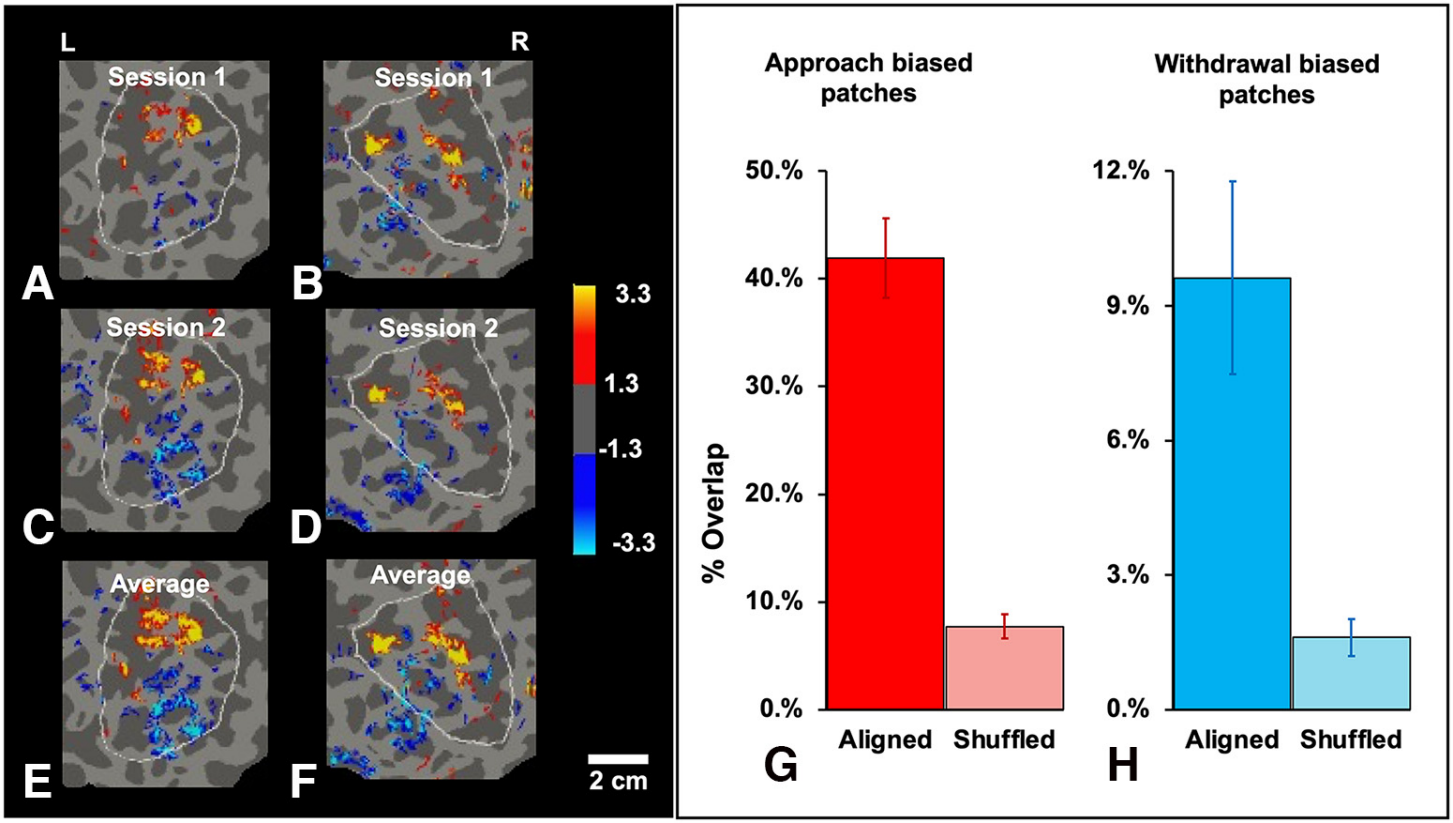
Consistency of evoked activity maps across sessions. Left panel, Consistency of BOLD activity maps in flattened cortex of the ROI, in response to the approaching versus withdrawing stimuli, across each of two sessions (***A–D***), and their average across both sessions (***E***, ***F***), from subjects 3 and 2 (left and right panels, respectively). In panels ***A–F***, yellow/red = approach > withdrawal; cyan/blue = withdrawal > approach. The corresponding scale on the right indicates -log10(p). Right panel, Overlap analysis confirms the consistency of the within-subject maps across scanning sessions. ***G***, Group-averaged (*n* = 7) level of overlap in within-subject maps of approach > withdrawal activity, when generated in session 1 compared to session 2, when the two maps were aligned correctly relative to each other and to the cortical topography (left bar, saturated red), and when pseudo-randomly misaligned in topography (“shuffled,” averaged over 1000 iterations; right bar, light red; *t* test: *p* < 0.0001). ***H***, Analogous levels of overlap in maps of withdrawal > approach activity, when aligned (left bar, saturated blue) and when misaligned (right bar, light blue; *t* test: *p* < 0.0001). Brackets represent 1 SEM.

Two individual subject maps are shown in Figure 4. Maps from all subjects showed similar, approach-biased patches the parietal ROI (Fig. 5). These maps appeared highly consistent within each subject/hemisphere when compared across sessions (Fig. 6*A–F*). These observations were supported by comparisons within a given subject, when the maps of the two sessions were topographically aligned (as *in vivo*), compared with the nonaligned (systematically shuffled) map comparisons (*p* < 0.0001; Fig. 6*G*,*H*). Thus, the approach-versus-withdrawal maps did not appear to be dominated by statistical noise, which should vary across sessions.

Within the parietal ROI, the specific topography of the evoked patches varied between subjects (see Fig. 5), akin to the idiosyncratic (fingerprint-like) mapping of cortical columns in macaque visual cortex, which also varies widely between individuals (Tootell et al., 1988; Horton and Hocking, 1996). Presumably, the idiosyncratically arranged patches in each individual subject were effectively “averaged out” in the group averaged maps here (Fig. 3) and previously (Holt et al., 2014), because the patches do not align with each other across subjects.

In addition to patches that showed approach-biased activity, the single subject maps often included patches that were more strongly activated by the withdrawing stimuli (Figs. 4, 5). Like the approach-biased patches, the locations of the withdrawal-biased patches remained largely consistent across scan sessions within a given subject (Fig. 6). However, in the parietal ROI, the number of these withdrawal-biased patches was significantly lower than the number of the approach-biased patches, above all thresholds between 10^−2^ through 10^−5^ (all *p* < 0.01).

### BOLD evidence for a columnar organization

These data raise the possibility that these approaching and withdrawing 3D face stimuli differentially activated a previously unknown set of functionally distinct cortical columns in posterior parietal cortex. If the evoked “patches” are indeed “columns,” and to the extent that BOLD activity faithfully reflects the 3D shape of neural activity, then these BOLD-based sites should be elongated along the radial axis (i.e., perpendicular to the cortical surface) within the gray matter. This radial consistency of a given functional property is a defining feature of cortical columns (Mountcastle, 1957; Hubel and Wiesel, 1962, 1968; Horton and Adams, 2005). To test for such a radial elongation, we first examined the single subject data qualitatively, then conducted a formal statistical analysis.

**Figure 7.**
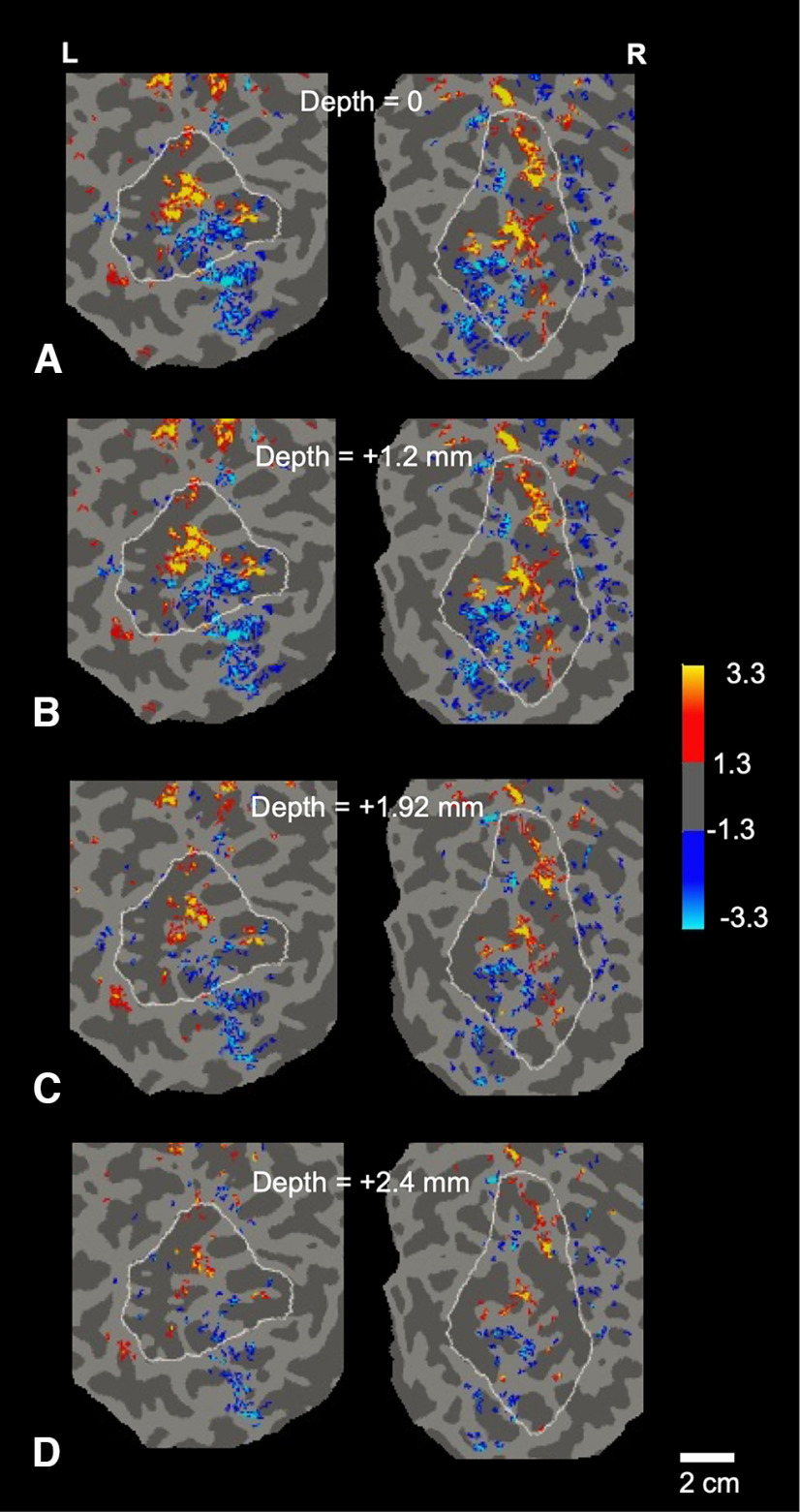
The patchy topography in the approach versus withdrawal maps remained consistent across variations in gray matter depth. Maps of approach versus withdrawal responses of the right and left hemispheres of subject 1 are shown across variations in cortical depth in flattened cortex, within the parietal ROI. ***A***, Activity map sampled from vertices centered on the cortical surface (depth 0 mm), which included activity in cortical layers 1 and 2 (and perhaps the pial vasculature). The activity in panel ***B*** was (on average) centered midway through the gray matter (depth = 50% gray matter), including cortical layer 4. The activity map in panel ***C*** was centered at a depth of 80% of the gray matter, including cortical layers 5 and 6. Panel ***D*** was located at the gray/white matter boundary (depth 100%), including activity in layer 6. The activation amplitude in panel ***D*** was reduced relative to panels ***A–C***, presumably because of partial volume inclusion of the white matter (which shows negligible BOLD variation). The significance scale on the right indicates -log10(p), yellow/red = approach > withdrawal; cyan/blue = withdrawal > approach. The boundary of the parietal ROI is indicated in each map by a white outline.

First, we observed that the flattened activity maps that were sampled preferentially from the upper cortical layers were strikingly similar to those sampled through middle and lower cortical layers (Fig. 7*A–D*), as in the well-established columns within visual area V2 (Nasr et al., 2016). In 3D, this observation suggests a radial elongation of the BOLD-evoked patches.

**Figure 8.**
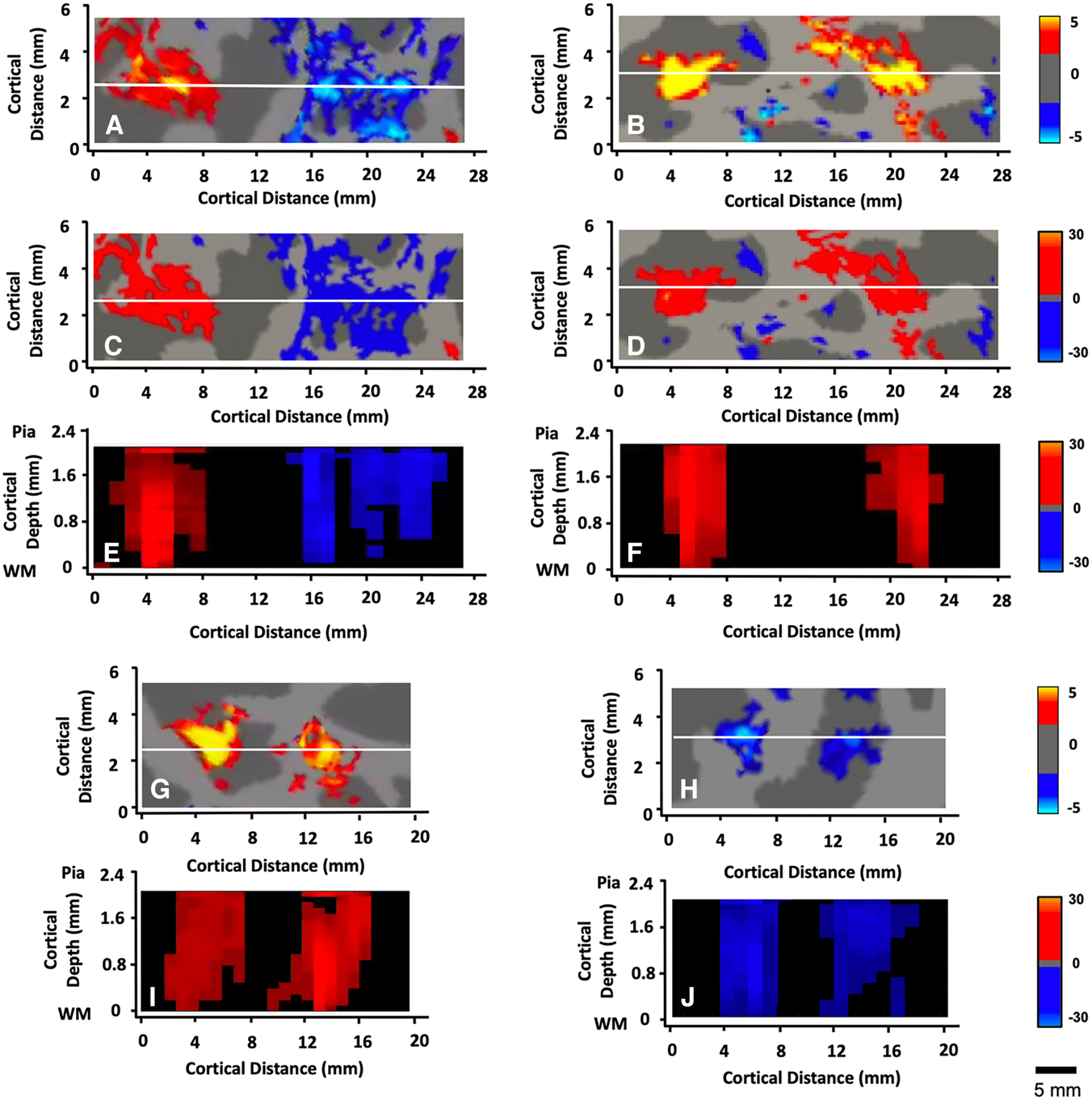
Activity evoked by the approaching (vs withdrawing) face stimuli is radially elongated. Flattened activity maps in the parietal ROI were sampled when centered at each of 20 equally spaced cortical depths, which were then radially aligned and reconstructed into a 3D map of the original cortex. Activity was then sampled along a line drawn across the flattened cortex (white line, ***A–D***). The activity in panels ***A–D*** was averaged across cortical depths (i.e., 10 through 90%), and shown in views analogous to classical views of cortical columns (***E***, ***F***). ***C***, ***D***, Same data as in ***A***, ***B***, except that values in the activity map were re-scaled to match the broader range of pseudo-color activity scaling in ***E***, ***F***. ***G***, ***H*** and ***I***, ***J***, Additional examples of the BOLD activity across variations in cortical depth, sampled from a different subject. Other details are as in ***A***, ***B*** and ***E***, ***F***, respectively. Generally, BOLD activity was elongated along the radial axis. Given the averaged gray matter thickness in this region (2.37 mm), and the voxel size (1.1 mm^3^, iso), the activity maps were nominally independent at the two depth extremes (10% and 90%). Activity at other depths may partially overlap, reflecting the relative depth levels and the local cortical thickness, plus the voxel size and the known spread of the BOLD signal. Panels ***A***, ***C***, and ***E*** are showing data from subject 5 (left hemisphere, thickness = 2.21 mm). Panels ***B***, ***D***, and ***F*** are showing data from subject 2 (right hemisphere, thickness = 2.46 mm). Panels ***I*** and ***G*** are showing data from subject 5 (right hemisphere, thickness = 2.28 mm). Panels ***H*** and ***J*** are showing data from subject 3 (right hemisphere, thickness = 2.41 mm). Yellow/red = approach > withdrawal; cyan/blue = withdrawal > approach.

Next, we sampled the evoked activity differences from a viewpoint perpendicular to the cortical surface, as in classic views of “columns” across the cortical layers (see Materials and Methods). These results (see Fig. 8) further supported the presence of a radial elongation of the evoked BOLD activity, in response to both approaching and withdrawing stimuli.

**Figure 9.**
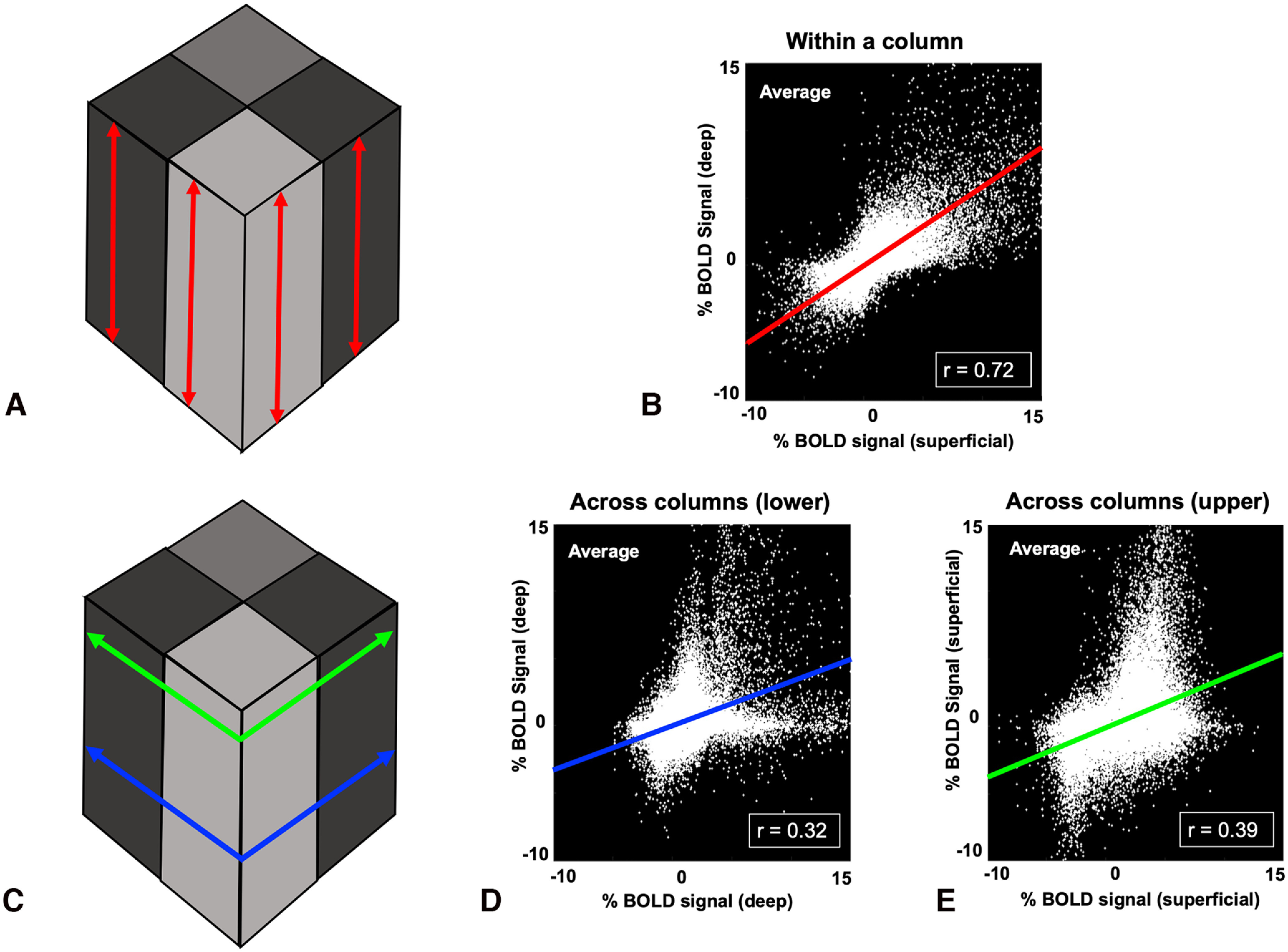
Correlations of evoked approach-bias versus withdrawal-bias BOLD responses sampled along radial-parallel versus surface-parallel axes in the flattened cortical surfaces within the parietal ROI. Evoked activity was sampled from all vertices within the gray matter, from all (*n* = 14) hemispheres. The sampling planes are schematized in panels ***A*** and ***C***. ***B***, Correlation of activity when sampled along an axis perpendicular (radial) to the cortical surface. ***D***, ***E***, Correlation values when sampled across a comparable distance in the perpendicular cortical plane, i.e., parallel with the cortical surfaces and layers, deep (depth = 0.9) in panel ***D***, and superficial (depth = 0.1) in panel ***E***. Functional response variations were more strongly correlated with each other when sampled along the radial axis, compared with the surface-parallel axis.

Finally, we measured the correlation between stimulus-driven BOLD activity (approach vs withdrawal) in the surface-normal (i.e., radial) versus surface-parallel (i.e., within-laminar) axes (see Materials and Methods). As a further control, two surface-parallel maps were tested: one at shallow cortical depths, and one at deeper depths (Fig. 9). As shown in Figure 9 and Table 1, all seven subjects showed correlation coefficients measured along the radial axis (perpendicular to the cortical surface) that were approximately twice the magnitude of the coefficients measured along the orthogonal plane (i.e., parallel with the cortical surface), in planes centered in both the upper and lower cortical depths/layers (*n* = 7, *t* = 16.82, *p* < 0.001; within a column vs across columns in superficial layers: *n* = 7, *t* = 11.38, *p* < 0.001).

**Table 1.** Correlations of functional activity along radial-parallel versus surface-parallel axes, in each subject

	Correlation coefficient within a column	Correlation coefficient across columns (deep layers)	Correlation coefficient across columns (superficial layers)
Subject 1	0.71	0.28	0.42
Subject 2	0.67	0.26	0.33
Subject 3	0.68	0.29	0.38
Subject 4	0.68	0.25	0.34
Subject 5	0.74	0.29	0.49
Subject 6	0.76	0.24	0.27
Subject 7	0.57	0.18	0.25
Average	0.72	0.32	0.39

Data (Pearson's r values) are averaged across both hemispheres, in each subject. In every subject, the functional response variations were more highly correlated in the radial axis, compared with either of the surface-parallel axes.

In fact, the differences in within-column versus across-column correlation strengths suggested an even stronger radial bias here in parietal cortex, compared with that shown previously in the well-established columns in visual area V2 (cf. Nasr et al., 2016, V2 in Fig. 7, compared with Fig. 9 and Table 1 here). Specifically, the average *z* scores in the current parietal cortex data measured in radial versus surface-parallel axes were 0.84 versus 0.26, respectively, whereas comparable *z* scores in the prior V2 data were 0.81 versus 0.51.

It is widely accepted that hemodynamic changes arising from sites located immediately “above” the gray matter (including the larger diameter vessels in the pia) contribute significantly to BOLD signal changes in scans using conventional gradient echo sequences at 3T (Yacoub et al., 2003, 2005; Duong et al., 2003). Although the techniques used here (e.g., 7T and small voxels) reduce such concerns (Merboldt et al., 2000; Triantafyllou et al., 2005; Polimeni et al., 2010; De Zwart et al., 2002), we nevertheless evaluated the extent to which the radially elongated BOLD responses might reflect hemodynamic effects arising outside the gray matter (e.g., in pia and neighboring spaces).

We measured BOLD activity in cortex across a range of cortical depths, both (1) within the gray matter (at conventionally-defined depths, “below” the cortical surface), and (2) at equal distances from the cortical surfaces in flattened planes, sampled “above” the cortical surface, as described elsewhere (J.E. Chen et al., 2021). Resultant surface-parallel BOLD variation was compared at “below,” and “above” the flattened cortical surface (respectively, in steps of 0, −1.2, −1.9, −2.4 mm depths below, and +1.2, +1.9, and +2.4 mm above) the cortical surface. Consistent with the maps in Figure 7, Table 2 showed significant topographical correlations between all four maps that were acquired within the gray matter (all Pearson *r* > 0.73, *p* < 0.001). However, we found significantly weaker correlations between any combination of the maps that were sampled below-the-surface versus above-the-surface (all *t* > 16.53, *p* < 0.0001). These results suggest that the topographical organization of the BOLD maps described in Figures 4–9 were not unduly dominated by BOLD signal from pial vessels. Given these data, and for the sake of brevity, below we refer to both the approach-selective and withdrawal-selective “patches” as “columns,” specifically termed P columns (i.e., presumptively related to PS).

**Table 2.** Correlation of functional activity below the cortical surface versus above the cortical surface

	A	B	C	D
Subject 1	0.82	0.19	0.12	0.12
Subject 2	0.81	0.10	0.05	0.05
Subject 3	0.80	0.15	0.09	0.09
Subject 4	0.80	0.11	0.07	0.06
Subject 5	0.82	0.22	0.15	0.14
Subject 6	0.84	0.43	0.26	0.21
Subject 7	0.73	0.10	0.07	0.07
Average	0.80	0.18	0.11	0.10
*t* test		*t* = 16.53 *p* < 0.001	*t* = 31.22 *p* < 0.001	*t* = 41 *p* < 0.001

The correlation coefficients (Pearson's r values) between radially-aligned BOLD variations spanning various cortical “depths.” For all of these correlations, the reference BOLD variation is sampled from the deeper layers in the gray matter (cortical depth = 1.92 mm). In A, the second value is derived from the cortical surface and superficial cortical layers (depth = 0 mm), reflecting BOLD variations within gray matter columns. Confirming prior data (Figs. 7, 9), the correlations were strong in all subjects (all *r* > 0.7). In B–D, the second value represents BOLD variation in cortical flattened planes that are sampled 1.2, 1.92, and 2.4 mm above the cortical surface, i.e., spanning the pia. The correlation coefficients in B–D were much lower than in A (all < 0.19), indicating that the topographic variation measured near-but-above the cortical surface did not match the topography of the activity-driven maps measured within the gray matter.

### Experiment 2: responses to stationary 3D face stimuli across a range of interpersonal distances

In the conventional SDP measurements [in both the classic version (Williams, 1971; Hayduk, 1983) and as adapted here for use in the scanner (in experiment 1)], the stimuli move continuously, either approaching or withdrawing. Prior studies in humans (Cléry et al., 2015; Candini et al., 2021) and nonhuman primates (Cléry et al., 2017) raise the possibility that these dynamic PS measurements reflect contributions from visual motion cues in addition to PS requirements per se, e.g., based on anticipation of the oncoming trajectory of the moving stimuli. On the other hand, it is also known that the discomfort evoked by PS intrusion at a given distance is largely time-insensitive, i.e., relatively constant over a wide range of intervals (from seconds to months) and over extensive repetitions (Hayduk, 1981a; Wormith, 1984; Tootell et al., 2021; Welsch et al., 2021; see also Fig. 2).

Thus, in experiment 1, it is unclear whether stimulus motion confounds conventional SDP measurements of PS. To address this question, experiment 2 measured fMRI responses to *stationary* face stimuli. The stationary faces (Fig. 1*B*) were presented at several virtual distances from the observer, spanning both near and far distances relative to each subject's PS boundary (9 distances total, including each subject's PS boundary), in pseudo-randomized order using an event-related design (see Materials and Methods). BOLD responses to the stationary stimuli were measured independently in the approach-biased and withdrawal-biased columns.

**Figure 10.**
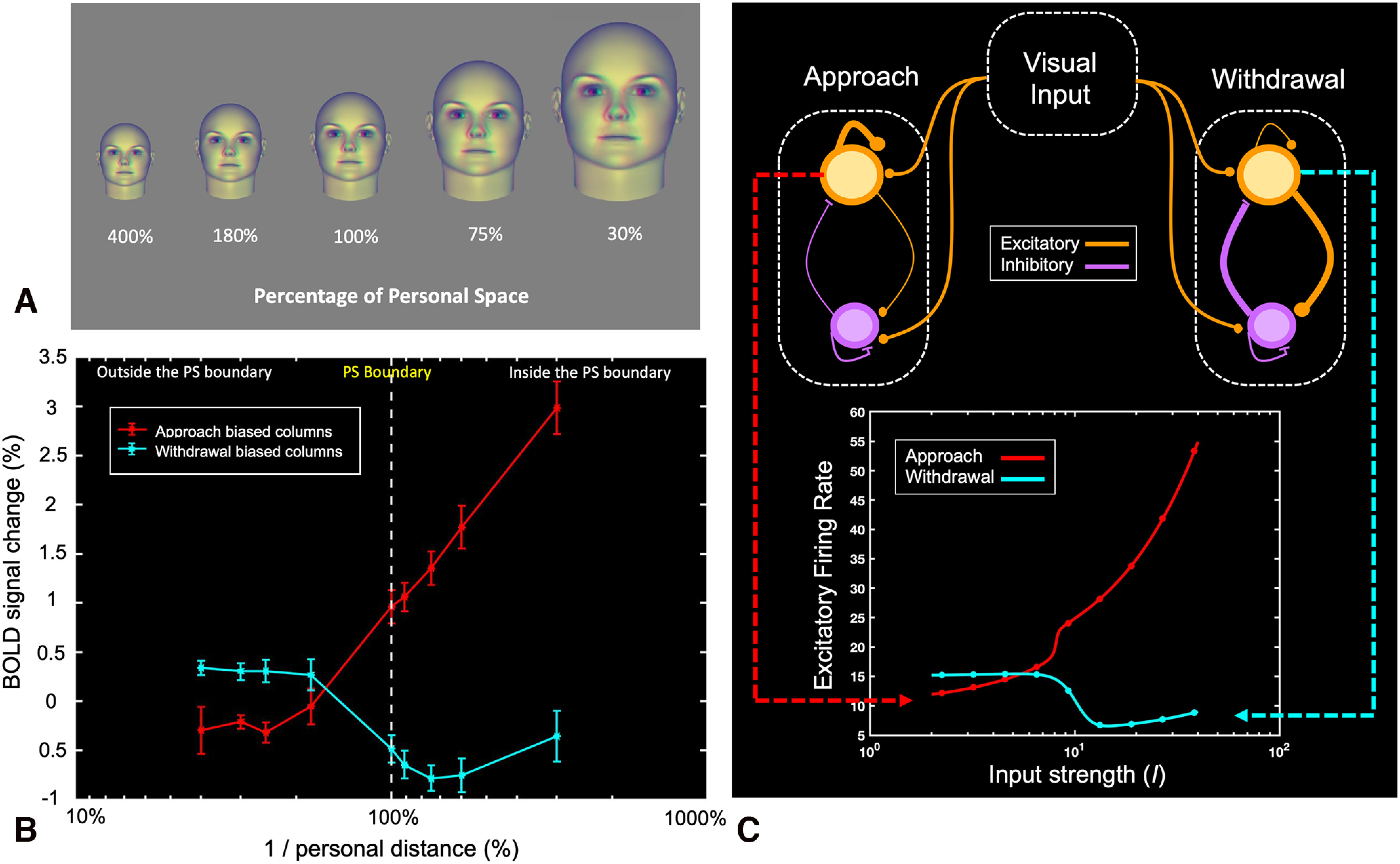
BOLD responses to variations in stationary interpersonal distance. In the scanner, subjects were presented with range of virtual face stimuli, of differing binocular disparity and size. The combination of size and disparity differences in the virtual faces (e.g., ***A***) matched those present in real-world faces at comparable viewing distances. ***B***, Average BOLD peak amplitude in response to each virtual distance. The response functions were measured independently in the approach-biased and withdrawal-biased columns. The vertical dashed white line indicates each subject's personal space boundary (100%); other distances were normalized, precalculated, and presented as percentages of that value, for each subject (see Materials and Methods). Error bars represent one standard deviation. ***C***, One possible computational scheme to generate such parietal responses (bottom) from visual cortical inputs (top). Results from experiments 1 and 2 suggested that the input corresponding to physically closer faces was larger in size, hence stronger. In this scheme, the average firing rates of the excitatory and inhibitory populations in inferior parietal cortex were represented by rate equations (Wilson and Cowan, 1972), in which each population received weighted inputs from the other population and itself, through a sigmoid nonlinearity (Table 3). For simplicity, this model assumed that BOLD levels represent the excitatory postsynaptic response, although it is known that BOLD signals reflect a combination of both presynaptic and postsynaptic activity (Logothetis et al., 2001; Logothetis, 2003). The synaptic weights between and within the two populations were considered free parameters, which were tuned to approximate the response profiles of approach-biased and withdrawal-biased columns (Table 3). In the approach-biased columns, the model suggested that a strong recurrent excitation and a weak recurrent inhibition could account for the activity profile of the excitatory population in response to the upstream visual input (***C***, top, orange and purple, respectively). Because of the weak recurrent inhibition, the response of the excitatory population increased in response to the input. When the input was strong enough to activate the excitatory population beyond its intrinsic soft threshold, there was a rapid increase in the excitatory firing rate because of the strong recurrent excitation (***C***, bottom, red). In the withdrawal-biased columns, a weak recurrent excitation and a strong recurrent inhibition could account for the response of the excitatory population to the input. For weaker inputs, inhibition dominated over excitation; hence the excitatory firing decreased as a function of the input. However, as the input became even stronger, the activity of the inhibitory population saturated and excitation overcame the inhibition, which eventually caused the excitatory firing rate to plateau (***C***, bottom, cyan).

The approach-biased columns showed a consistent pattern of BOLD responses to face variations across corresponding variations in virtual distance (Fig. 10*A*). The mean BOLD response to faces presented at the four largest distances (i.e., “further” than the PS boundary) showed a negligible (baseline) response (all *p*-values > 0.16), i.e., essentially nonresponsive to the virtually “far” face stimuli. In contrast, BOLD responses to faces presented closer than the PS boundary increased steeply and monotonically, such that the highest response was evoked by the closest face. The withdrawal-biased columns showed a less dramatic response function to the stationary faces, which was nevertheless largely inverted in sign relative to baseline, and negligible at distances further than the PS boundary (Fig. 10*B*).

**Table 3.** Parameters of the computational model

Parameter	Description	Approach	Withdrawal
$r_{E}$	Excitatory (E) firing rate		
$r_{I}$	Inhibitory (I) firing rate		
$W_{EE}$	E-to-E synaptic weight	5	0.1
$W_{EI}$	I-to-E synaptic weight	−0.1	−10
$W_{IE}$	E-to-I synaptic weight	0.1	10
$W_{II}$	I-to-I synaptic weight	−1	−0.1
$\theta_{E}$	E baseline input	10	10
$\theta_{I}$	I baseline input	10	1
$W_{E}$	Input synaptic weight to E	1	0.1
$W_{I}$	Input synaptic weight to I	10	1
θ	Neural threshold	20	
I	Input		

**Figure 11.**
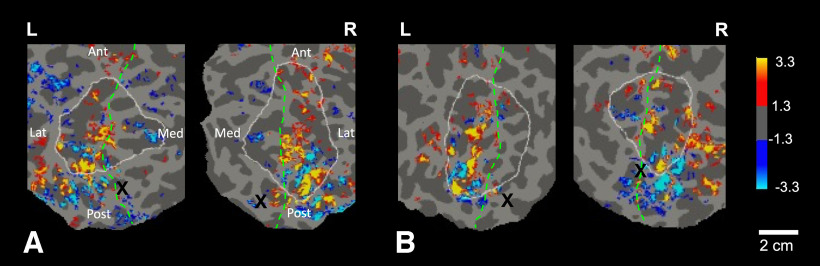
Individual maps of near- and far-selective disparity columns (red-yellow and blue-cyan, respectively, scale bar on the right), in response to visual presentation of random dot stereograms in Experiment 3. Panels show flattened maps of cortical activity, in both hemispheres, in each of two individual subjects (1 and 3) in ***A*** and ***B***, respectively. In both A and B, the medial-lateral border (where the cortex folds) is shown as a dashed green line, and the parietal ROI is indicated by a white outline. As expected, disparity columns were also evident within neighboring visual cortical area V3A, indicated here with black Xs. Ant, anterior; Post, posterior; Med, medial; Lat, lateral; L, left hemisphere; R, right hemisphere. The significance scale on the right indicates -log10(p).

Numerical values of the parameters in a computational scheme in which visual inputs are combined to mimic personal space coding (Fig. 11B). Following the widely used framework of the firing rate neural models (Wilson and Cowan, 1972), the firing rates of excitatory and inhibitory populations were modeled as the following:
$$\frac{dr_{E}}{dt}=-r_{E}+W_{EE}f\left(r_{E}\right)+W_{EI}f\left(r_{I}\right)+\theta_{E}+W_{E}I$$
$$\frac{dr_{I}}{dt}=-r_{I}+W_{IE}f\left(r_{E}\right)+W_{II}f\left(r_{I}\right)+\theta_{I}+W_{I}I,$$ where $r_{E}$ and $r_{I}$ are the firing rate of excitatory and inhibitory populations, respectively. $W_{EE}$ is the synaptic strength of excitatory-to-excitatory connections, $W_{EI}$ is the strength of inhibitory-to-excitatory connections, $W_{IE}$ is the strength of excitatory-to-inhibitory connections, and $W_{II}$ is the strength of inhibitory-to-inhibitory connections. Each population has a baseline activity represented by $\theta_{E}$ and $\theta_{I}$, respectively. Finally, each population receives the external input I weighted by synaptic strengths $W_{E}$ and $W_{I}$, respectively. Each population receives the firing rate of the other population as input through a sigmoid nonlinear function f(x)=1/(1+exp(−(x+θ))), where θ represents the intrinsic soft threshold of the population activity. The variable of interest in these simulations was the stable steady-state firing rate of the excitatory population as a function of the strength of the external input I.

### Modeling of approach and withdrawal biased columns

It has been reported that intrusion into PS evokes responses in the amygdala (Kennedy et al., 2009; Todd and Anderson, 2009; Wabnegger et al., 2016; see also Nacewicz et al., 2006), consistent with long-standing evidence that the amygdala is involved in the processing of social threat and avoidance (Klüver and Bucy, 1937; Breiter et al., 1996; Emery et al., 2001; Zald, 2003; Mason et al., 2006; Adolphs, 2008; Adolphs, 2010). Thus, it is possible that input from the amygdala influences the parietal cortical responses to stimulus distances that are closer (but not further) than each subject's PS boundary, as in the function shown in Figure 10*B*.

Alternatively, we hypothesized that a differential weighting of purely visual inputs could instead account for these response differences in parietal cortex. To evaluate the computational feasibility of this idea, we tested a scheme in which both approach-selective and withdrawal-selective population responses resulted from ascending (“upstream”) input from visual cortex. This model matched the experimental results well (Fig. 10*B*,*C*). Thus, the differing response profiles of the approach-biased and withdrawal-biased columns observed here could reflect correspondingly differing strengths of the local excitatory and inhibitory synaptic signals from visual cortex to each column type in parietal cortex, without requiring input from the amygdala. Further testing is necessary to validate this model.

### Experiment 3: near-selective and far-selective columns based on binocular disparity

As in real life conditions, the binocular disparities across the face stimuli of experiments 1 and 2 varied with distance from the viewer. Thus, it might be asked whether the approach-biased and withdrawal-biased columns involve disparity-selective neurons and columns similar to those described previously in occipital visual cortex in macaques (Felleman and Van Essen, 1987; Poggio et al., 1988; Roy and Narahashi, 1992; Deangelis et al., 1998; Deangelis and Newsome, 1999; Adams and Zeki, 2001; Tanabe et al., 2005; G. Chen et al., 2008) and in humans (Goncalves et al., 2015; Nasr et al., 2016; Tootell and Nasr, 2017). However, this hypothesis presumes that “near” and “far” disparity-selective columns extend beyond occipital cortex, anteriorly into parietal cortex, and no such disparity columns have been reported previously beyond occipital (visual) cortex.

**Figure 12.**
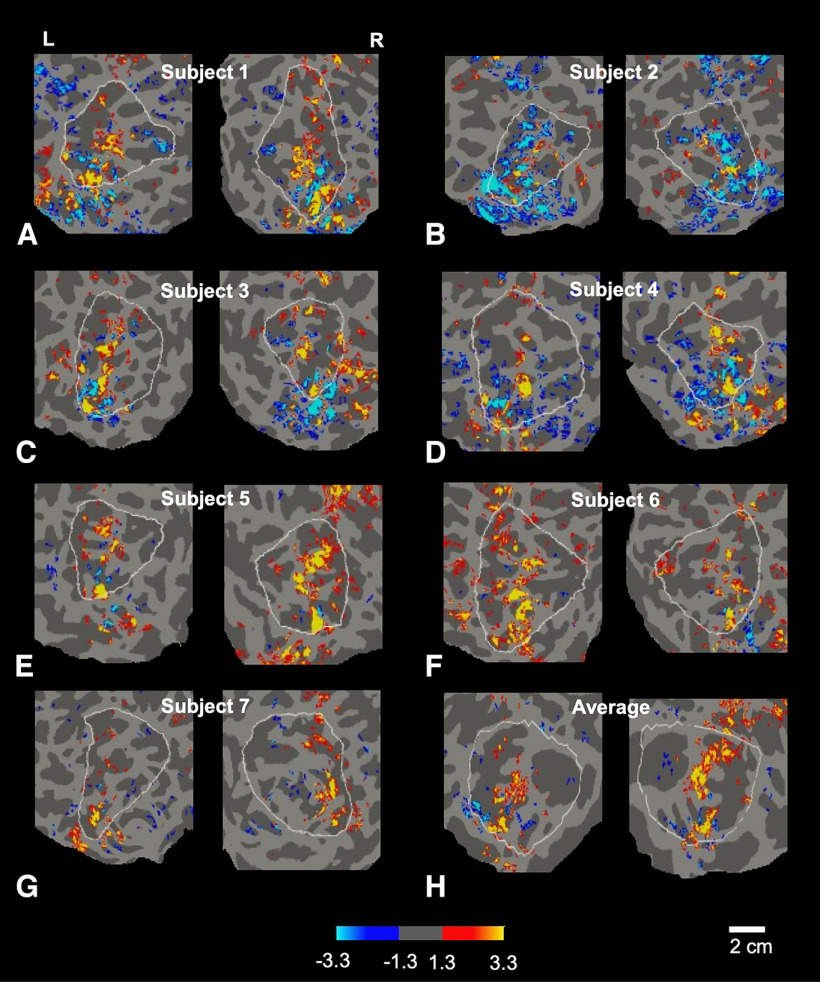
Maps of binocularly fused disparity-selective activity in each subject, in both hemispheres, evoked by binocularly viewed random dot arrays. Higher activity to visually near and far distances is shown in red-yellow and blue-cyan, respectively. All maps were averaged across both scan sessions, in each individual subject, in both right and left hemispheres (panels ***A–G***), in the parietal cortex region of interest (see white boundary in each map). Panel ***H*** shows the average activity across all subjects. The significance scale indicates –log10(p).

Accordingly, we next tested for the presence of disparity columns in the posterior parietal ROI, using conventional random dot stereogram stimuli that selectively activated near-distance and far-distance disparity-selective columns in previous fMRI studies of occipital cortex (Tsao et al., 2003; Nasr et al., 2016; Nasr and Tootell, 2018a, 2020). When disparities in the two monocular images were binocularly fused, this random dot arrays produced a stereoscopic percept of multiple 3D cuboids, moving continuously through near or far visual depths (see Fig. 1*C*; and Materials and Methods). All subjects confirmed this stereoscopic percept during the scan session. The corresponding near-disparity and far-disparity patches extended bilaterally into posterior parietal cortex (Figs. 11, 12), but few disparity patches were found further anterior in parietal cortex. This is consistent with our overall hypothesis that posterior parietal cortex functions as a “transition” area, including both sensory and egocentric spatial encoding.

Comparisons across maps sampled at different cortical depths suggested that these disparity-selective BOLD sites in parietal cortex are radially elongated (i.e., columnar), consistent with the organization of disparity-selective columns identified in macaque monkeys and in high field fMRI studies in human occipital cortex. Accordingly, we refer to these disparity-sensitive patches in parietal cortex as D (disparity) columns.

In the parietal ROI, the near-selective disparity columns were more numerous than the far-selective disparity columns, across the testable range of thresholds (above all thresholds between 10^−2^ through 10^−5^; *t* test: all *p*-values < 0.01). This bias for near- (relative to far-) selective columns in parietal cortex may be related to a near-disparity versus far-disparity bias reported in neighboring occipital cortex (Nasr and Tootell, 2020). Overall, we conclude that posterior parietal cortex includes visually driven, stereo-selective columns, similar to those previously described in occipital cortex.

### Topographical relationship between column types

Next, we investigated how the P and D column types are distributed relative to each other. Initial observation of the maps (compare Figs. 4, 5 and 11, 12) suggested that the two categories of columns (P and D), and/or the two functional poles in each of these categories (approaching vs withdrawing, and near vs far), might have a nonoverlapping relationship, analogous to the interdigitation of thin versus thick type columns demonstrated in human visual cortical areas V2 and V3 (Nasr et al., 2016; Dumoulin et al., 2017; Tootell and Nasr, 2017). However, alternative topographic relationships are possible, including: (1) a random organization of the two columnar categories and poles, or (2) an overlap of the two columnar categories and/or poles (i.e., only one set of columns, which respond to all of our test stimuli, to varying extents).

By definition, two of the possible stimulus contrasts (near vs far disparity, and approaching vs withdrawing) are nonoverlapping relative to each other, based on the nature of the fMRI subtraction per se. Thus, we examined the relationship between maps evoked by the four remaining functional contrasts (near disparity vs approach, far disparity vs withdrawal, near disparity vs withdrawal, and far disparity vs withdrawal). BOLD response levels were measured in each vertex within each hemisphere, in response to these four functional contrasts, when the maps were: (1) accurately aligned (as *in vivo*), compared with (2) computationally “shuffled” (pseudo-randomly misaligned).

**Figure 13.**
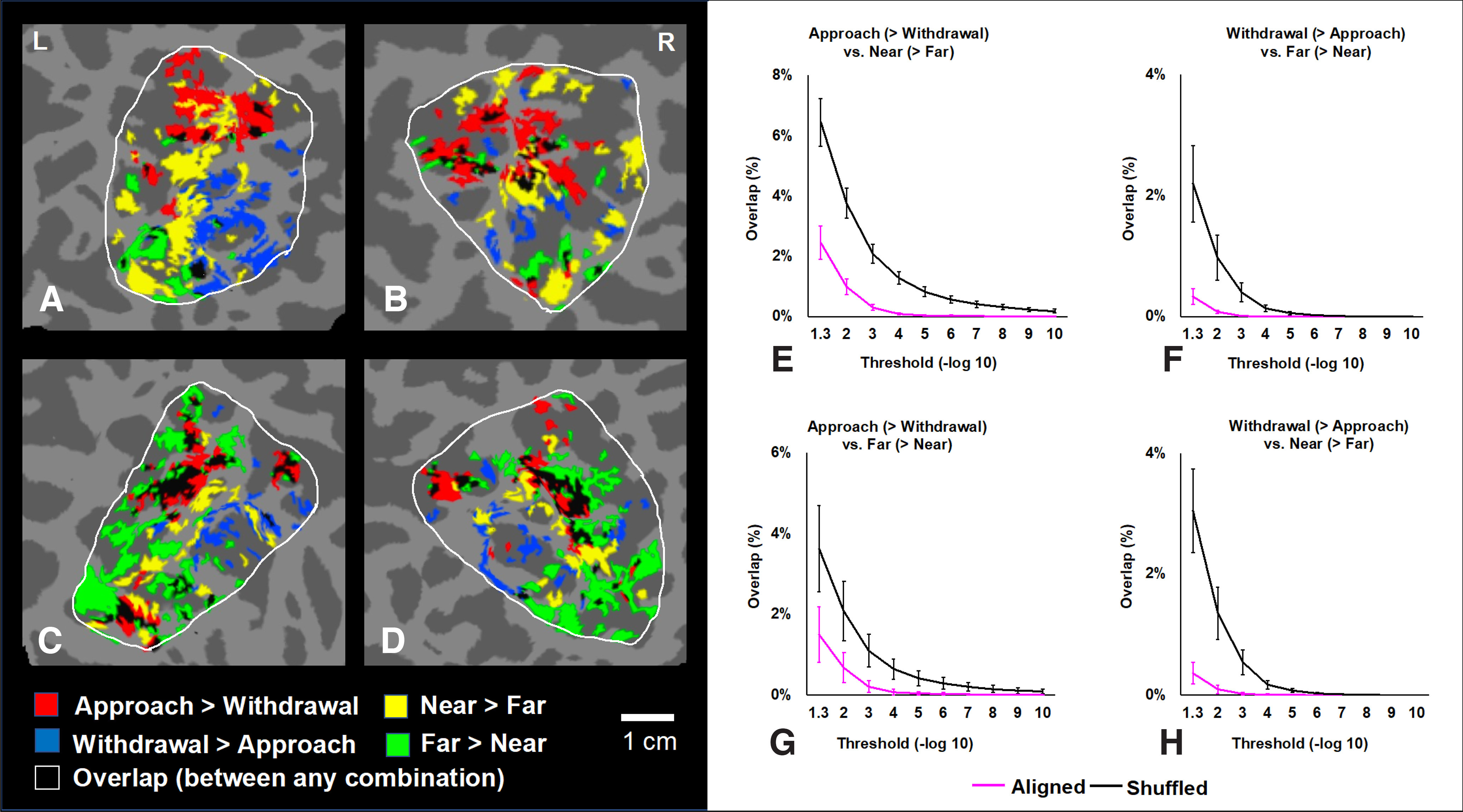
Topographic relationship of P and D columns. ***A–D***, Maps of the cortical overlap (and nonoverlap) in the flattened activity maps of approach-biased and withdrawal-biased activity, plus the activity in response to near-biased and far-biased binocular disparity, based on a colorized overlay of individual activity maps. Panels ***A*** and ***B***, and panels ***C*** and ***D***, show the activity of the left and right hemispheres of subjects 7 and 2, respectively, within the parietal ROI (indicated by white outlines). In all four maps, vertices that showed significantly biased activity to any single one (but not two or more) of the four functional contrasts are color-coded as shown below panels ***C*** and ***D***. Vertices that showed overlap (i.e., to two or more) of any of these functional contrasts are shown in black. The significance threshold for all contrasts was *p* < 0.05, uncorrected, to allow as much overlap as statistically possible. These examples suggest that there is relatively little overlap (black), implying a systematic segregation of function. In analyses across subjects, panels ***E–H*** show the extent of overlap in the maps, when averaged across all hemispheres (*n* = 14), within the parietal ROI, for all four pairwise contrasts of interest, across a range of activity thresholds (*x*-axis). The magenta line shows the extent of overlap when the maps were correctly aligned (as *in vivo*), across different thresholds (*x*-axis). As a control condition, the black line shows the level of overlap when the maps were aligned pseudo-randomly relative to each other, averaged across 1000 randomizations. Overlap is defined as the percentage of overlapped vertices (i.e., the number of vertices showing selectivity for a given functional contrast, divided by the number of vertices within the parietal ROI). Consistent with the examples in panel ***A–D***, these group-averaged results confirm a strong tendency toward interdigitation (e.g., segregated functional sensitivity) between all four of the functional contrasts tested here.

**Figure 14.**
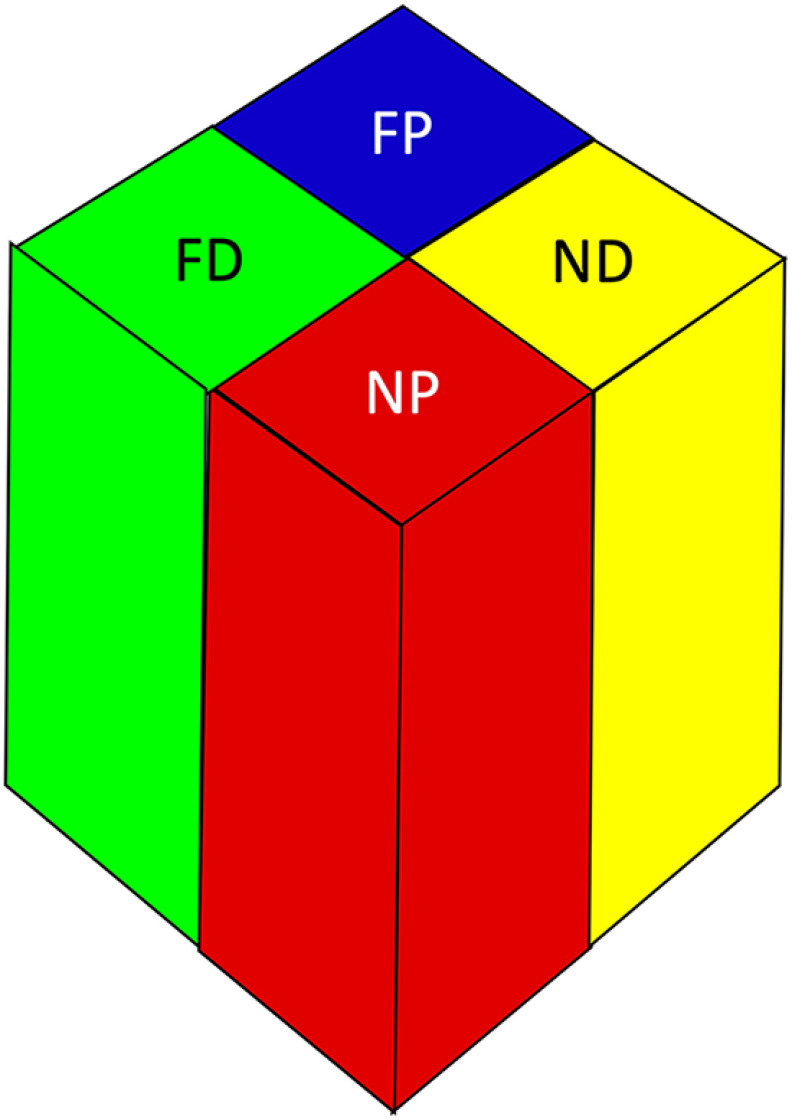
Hypothetical column-scale distance processing unit in parietal cortex. The cortical surface is represented at the top of the schematic and there are four types of radially-extending columns. ND = near disparity; FD = far disparity; NP = near personal; FP = far personal.

The results confirmed that BOLD activity peaks in all four paired stimulus contrasts were preferentially nonoverlapping (i.e., interdigitated; Fig. 13). Based on these results, one possible cortical processing “unit” is illustrated in Figure 14.

## Discussion

Overall, our evidence suggests that parietal cortical responses to variations in stimulus distance from the body are organized into at least two types of functional columns. Thus, one fundamental organizational feature of early visual cortex (i.e., functional segregation within multiple types of columns) apparently also exists in human parietal cortex.

### Encoding of interpersonal distance

More specifically, our results suggest that specific columns in the parietal cortex respond preferentially to variations in visually mediated virtual distance from an observer, along two stimulus dimensions: (1) purely visual (i.e., binocular disparity), and (2) partly nonvisual, presumptively related to interpersonal distance. In both dimensions, the activity maps differed significantly in response to presentations of visual stimuli at near versus far distance extremes.

However, the apparent congruence of “near” and “far” terms may be oversimplified, to the extent that the two underlying dimensions differ from each other. For instance, psychophysical sensitivity to stereoscopically-fused binocular visual stimuli ranges from a “near” limit roughly 10–20 cm from the eyes (depending on measurement technique; for review, see Wilcox and Allison, 2009) through a “far” limit at optical infinity. By comparison, the visual sensitivity to variations in personal space varies from a near limit close to the observer's skin, through a far limit averaging near 80 cm (for review, see Hayduk, 1983). The midpoint (zero-crossing) distance values between the near and far extremes of the two stimulus dimensions also differ from each other. In the dimension of personal space, those zero-crossing values (between near and far) are unrelated to where the subject is looking. However, based on visual binocular disparity, the zero-crossing distance depends on where the subject's eyes are converged, in each glance.

To simplify the experimental comparison, we studied both personal space cues and disparity cues by varying them along a common line of sight. However, sensitivity to personal space is not limited to visual stimuli. For instance, prior studies (Hayduk, 1981b; Wormith, 1984; Bogovic et al., 2016; Hecht et al., 2019) suggest that personal space surrounds the body, generating responses to intruders both visible and invisible. Auditory stimuli can also evoke discomfort when the source is perceived as near or within personal space (Lloyd et al., 2009; Noel et al., 2018). It has even been reported that proximity-driven discomfort can be evoked by simple statements that someone is within a subject's personal space (Wormith, 1984; Kennedy et al., 2009), without any manipulation of sensory cues. Consistent with this behavioral evidence, neurobiological studies have shown that sites in parietal cortex can be driven by proximity-related cues encoded by multiple senses, in addition to vision (Andersen, 1997; Holmes and Spence, 2004; Yang et al., 2011; Blanke, 2012; Ehrsson, 2012; Murray and Wallace, 2012; Sereno and Huang, 2014; Grivaz et al., 2017).

Similar to the overall behavioral response to personal space variation, the approach-biased columns in parietal cortex showed stronger BOLD responses to progressively nearer faces, even when such faces were stationary. Thus, based on their responses to both moving and stationary face stimuli, this category of columns could equally well be termed either (1) “approach-biased or withdrawal-biased,” or (2) “interpersonally-near or far-biased,” respectively. Our preferential use of the former term was largely arbitrary.

### Potential relationship to vasculature-related responses

It might be argued that our BOLD-based maps reflect variations in oxygenation in the radially arranged “diving” veins and arteries, rather than a columnar organization of neural responses per se. However, prior studies that directly compared the location of the radial vessels with cortical functional maps found very little (Keller et al., 2011) or no (Adams et al., 2015) topographical relationship between the two radial features (but see Zheng et al., 1991; Shmuel et al., 2010).

In addition, this interpretation is unlikely because the radial vessels (both arteries and veins) vary in diameter (between 20 and 150 μm), partly as a function of maximum cortical depth (Duvernoy et al., 1981), whereas the topographic diameter of BOLD-defined columns described here and elsewhere is much larger than 150 μm. Thus, each BOLD-defined column likely contained multiple radial vessels (i.e., not just one radial vessel in the middle of each column, as was initially speculated). However, partly because the range of capillary diameters partially overlaps that of radial vessels (Lauwers et al., 2008), radial veins could not be unambiguously distinguished from capillaries using current techniques. Another complication is that the reperfusion territory of diving veins is conical in shape, rather than strictly radial (Duvernoy et al., 1981).

More conceptually, it seems unlikely that different sets of BOLD-based columns (e.g., a functional interdigitation, as found here and previously; Nasr et al., 2016; Dumoulin et al., 2017; Tootell and Nasr, 2017) could arise from any single common vascular substrate. Ultimately, we conclude that our BOLD-based “columns” likely include (but are not dominated by) radially aligned vessels.

### Relationship to prior findings in macaques and humans

Our column-scale fMRI findings in human parietal cortex are consistent with findings of some single neuron recordings in macaque parietal cortex. For instance, a bias for approaching stimuli has been reported in electrophysiological responses in macaque parietal cortex (Colby et al., 1993; Graziano and Cooke, 2006), and in areas that provide input to parietal cortex (Albright, 1989). Additional studies reported a preference for “near” visual stimuli in monkey parietal cortex (Colby et al., 1993; Duhamel et al., 1998; Hadjidimitrakis et al., 2011; Bremmer et al., 2013; Cléry et al., 2017).

Here, a discrete site within parietal cortex (dorsal parietal-occipital sulcus, dPOS) showed particularly strong BOLD responses to approaching and near faces. Consistent with this, Quinlan and Culham (2007) identified dPOS as an area that responded strongly to physically near (vs far) visual objects in humans. However, those authors ultimately concluded that dPOS responds selectively to inward vergence, rather than to near visual objects per se. However, that interpretation cannot fully explain the current results, in which the attention task required subjects to maintain a single vergence angle on the stimulus screen throughout the functional scans. Further research is necessary to fully understand the functions of this area.

### Columns in parietal cortex

More than 25 sets of functional columns have been reported in multiple areas in primate visual cortex. However, previously there has been little evidence for the existence of functional columns beyond visual cortex (but see Bruce et al., 1985). Insofar as the current P columns encode interpersonal social influences, the function of these parietal columns may be considered associative, rather than purely sensory. It is possible that earlier BOLD experiments evoked activity in higher order cortex that was radial in 3D shape, but not identified as columnar at that time.

Advanced fMRI techniques have revealed a coarse retinotopic organization in human parietal cortex (Swisher et al., 2007; Arcaro et al., 2011; Huang et al., 2017). Here, we could not identify any consistent topographical relationship between the current results and the retinotopy.

It is known that parts of parietal cortex (and cortical areas F4/5) can contribute to responses to approaching stimuli which are potentially physically threatening. The current data highlight that this role in threat processing may extend to stimuli that are socially threatening, or at least those that elicit arousal and/or attention. Additional brain areas that might be engaged by such stimuli include: (1) visual cortical sites involved in recognition of objects, and (2) parietal and premotor cortical areas involved in preparation of defensive actions.

### Clinical and public health implications

Abnormalities in personal space regulation have been observed in several neuropsychiatric disorders, including schizophrenia (Horowitz et al., 1964; Duke and Mullens, 1973; Srivastava and Mandal, 1990; Nechamkin et al., 2003; Park et al., 2009; De La Asuncion et al., 2015; Holt et al., 2015; Zapetis et al., 2022) and autism (Gessaroli et al., 2013; Kennedy and Adolphs, 2014; Lough et al., 2015; Perry et al., 2015; Asada et al., 2016; Candini et al., 2017; Noel et al., 2017; Mul et al., 2019). Given our findings, it is possible that early changes in the organization of the parietal cortex (perhaps including column-scale effects) are associated with the abnormalities in personal space regulation observed in these neurodevelopmental conditions. Future studies can test this possibility.

Although personal space preferences tend to be highly stable over time within individuals, some plasticity of personal space has also been observed. For example, tool use may lead to temporary expansion of personal space boundaries (Iriki et al., 1996; Berti and Frassinetti, 2000; Maravita and Iriki, 2004; Cardinali et al., 2009; Iachini et al., 2014; Biggio et al., 2017; Quesque et al., 2017; but see Patané et al., 2016). Also, recent studies raise the possibility that preferred interpersonal distances changed during the COVID-19 pandemic, perhaps because of social distancing mandates and/or fear of infection (Iachini et al., 2021; Serino et al., 2021; Welsch et al., 2021; Holt et al., 2022). It is not yet known whether such environmental factors affect personal space processing at the spatial scale of cortical columns.
